# Cold-based glaciation of Pavonis Mons, Mars: evidence for moraine deposition during glacial advance

**DOI:** 10.1186/s40645-020-0323-9

**Published:** 2020-03-12

**Authors:** Reid A. Parsons, Tomohiro Kanzaki, Ryodo Hemmi, Hideaki Miyamoto

**Affiliations:** 1 University Museum, The University of Tokyo, 7 Chome-3-1 Hongo, Bunkyo, Tokyo, 113-0033 Japan; 2 Earth and Geographic Sciences, Fitchburg State University, 160 Pearl St., Fitchburg, MA 01420 USA; 3 Department of Systems Innovation, The University of Tokyo, 7 Chome-3-1 Hongo, Bunkyo, Tokyo, 113-0033 Japan

**Keywords:** Thermomechanical ice sheet model, Mars, Amazonian climate, Moraine deposition, Tharsis, Glaciation

## Abstract

The three large volcanoes in the Tharsis region of Mars: Arsia, Pavonis, and Ascraeus Montes all have fan-shaped deposits (FSDs) on their northern or western flanks consisting of a combination of parallel ridges, knobby/hummocky terrain, and a smooth, viscous flow-like unit. The FSDs are hypothesized to have formed in the Amazonian during a period of high spin-axis obliquity which redistributed polar ice to the equatorial Tharsis region resulting in thick (> 2 km), flowing ice deposits. Based on previous ice flow simulations and crater surveys, the ridges are interpreted to be recessional drop moraines formed as debris on the ice sheet surface was transported to the ice margin—forming a long ridge sequence over an extended (∼100 Myr) period of ice sheet retreat. We test this hypothesis using a high-resolution, thermomechanical ice sheet model assuming a lower ice loss rate (~ 0.5 mm/year) than prior work based on new experimental results of ice sublimation below a protective debris layer. Our ice flow simulation results, when combined with topographic observations from a long sequence of ridges located interior of the Pavonis FSD, show that the ridged units were more likely deposited during one or more periods of glacial advance (instead of retreat) when repetitive pulses (approx. 120 kyr periodicity) of ice accumulation during high obliquity produced kinematic waves which advected a large volume of surface debris to the ice margin. If ridge deposition does occur during glacial advance, it could explain the cyclic pattern of ridge spacing and would link the dominant, 120 kyr periodicity in obliquity to the time interval between adjacent ridges. By measuring the spacing between these ridges and applying this timescale, we constrain the velocity of glacial margin to be between 0.2 and 4 cm/Earth year—in close agreement with the numerical simulation. This re-interpretation of the FSD ridged unit suggests that the timescale of FSD formation (and perhaps the duration of the Amazonian high obliquity period) was shorter than previously reported.

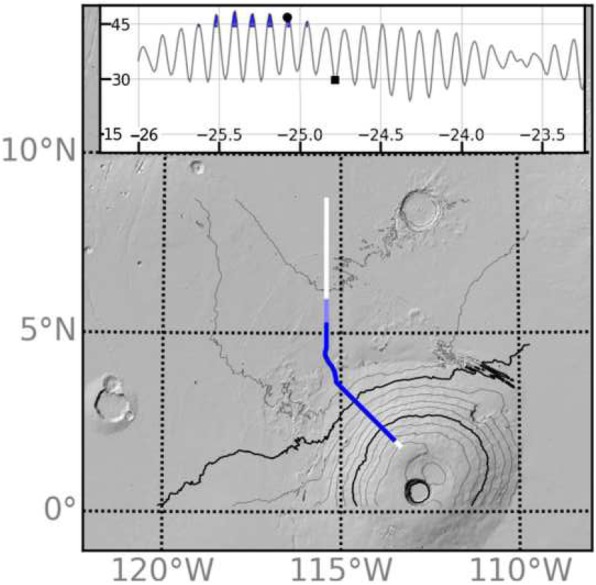

## Introduction

**Fig. 1:**
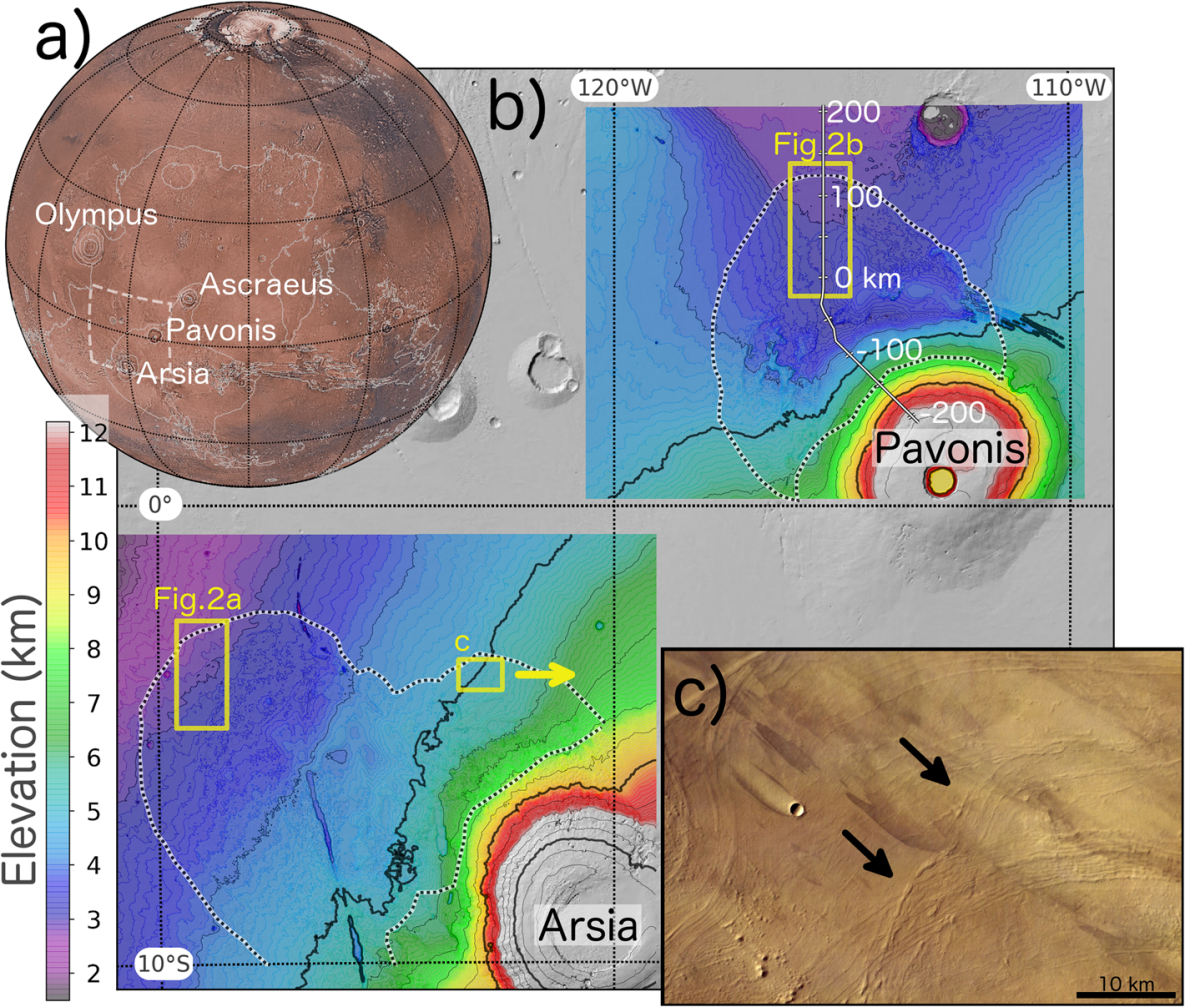
Topography of the Arsia and Pavonis FSDs. **a** A true color image mosaic from Viking data of the western hemisphere of Mars. **b** Detailed elevation (colored regions) of the FSDs (outlined with black/white dotted line) indicate rougher topography within the FSDs and presence of volcanic rifts at Arsia. White line with labeled distances gives the location of the flowline used in numerical model. **c** HRSC image of the outer margin of the Arsia FSD where the intersection of moraine-like ridges (arrows) indicates that glacial activity was not capable of eroding previous ridges during subsequent glacial advance

The fan-shaped deposits (FSDs) on the Tharsis volcanoes (Arsia, Pavonis, and Ascraeus) (Fig. 1a) were first described in detail using images from the Mariner 9 and Viking missions (Zimbelman and Edgett 1992). The outer margin of FSDs consists of a series of parallel ridges which have a lobate shape when viewed from above (black and white dotted line, Fig. 1b). A combination of smooth and knobby units is generally found in the interior of the FSDs which are most prominent on Arsia and Pavonis Mons. Early research efforts invoked various processes for FSD formation including gravity-driven landslides (Carr et al. 1977; Scott and Tanaka 1986), glaciation (Lucchitta 1981; Williams 1978), and pyroclastic volcanism (Zimbelman and Edgett 1992). Later support for glaciation of the Tharsis Montes came from the prediction from climate models of ice accumulation during times of high Martian spin-axis obliquity (Forget et al. 2006). During periods of high obliquity, the poles receive more insolation, and water ice sublimates in numerical simulations by Jakosky and Carr (1985). This water vapor is then transported equator-ward where orographic lifting and adiabatic cooling of air masses moving over the Tharsis volcanoes results in the precipitation and accumulation of ice in numerical climate models (Forget et al. 2006). The geologic observations and climate model results were combined in a study by (Fastook et al. 2008) to constrain the likely obliquity history which allowed these deposits to accumulate, attain their maximum extent, and retreat in accordance with crater age dates for the deposits (which indicate an exposure age of ∼300 Myr).

In this study, we investigate the influence of time-varying obliquity on the ice margin flow velocity, location, and shape using the thermomechanical Parallel Ice Sheet Model (PISM). Our simulation is compared with the observed spacing and cross-sectional area of ridges found within the Pavonis FSD in order to determine whether moraine deposition is more likely to occur during advance or retreat of the ice sheet. We first summarize the prior work regarding the FSD ridged unit and its geologic interpretation. Next, we describe the size and location of ridges at Pavonis Mons using CTX images and DEMs generated from CTX stereo pairs at both Pavonis and Arsia Montes. In the following section, we provide a description of the numerical model, its inputs, and assumptions. The final sections compare the simulation results with the observations, discuss the process(es) responsible for ridge emplacement, and summarize our findings.

### Prior geologic interpretation

The arcuate, distal portion of the FSDs generally consists of a series of parallel ridges (∼1 km spacing) with variable heights ranging from 5 to 50 m based on MOLA laser altimetry observations conducted by Head et al. (2003) at Arsia Mons. The delicate draping of ridges over underlying features (craters and lava flows) without modification (Head and Marchant 2003) is a characteristic shared by cold-based drop moraines in Antarctica (Bockheim 2010; Bockheim 2002; Marchant et al. 1994; Staiger et al. 2006). The regularity of ridge spacing and long lateral extent contrasts with landslide deposits found surrounding Olympus Mons (Francis and Waadge 1983) and contrasts with ridges deposited from turbulent pyroclastic flows (Head and Marchant 2003).

Geologic mapping efforts in the Tharsis region have made use of crater abundances and superposition relationships to conclude that the FSD formed in the late Amazonian and were likely contemporaneous with the most recent volcanic activity on both the volcanic edifices and radial rift zones of Arsia, Pavonis, and Ascraeus Mons. Landforms such as tuyas and tiered, mounded, leveed and digitate lava flows, which are indicative of lava-ice interaction, have been found in all three FSDs (Kadish et al. 2008; Scanlon et al. 2014; Scott et al. 1998; Shean et al. 2005).

The most recent crater density measurements for Arsia, Pavonis, and Ascraeus give best-fit ages of 210, 125, and 220 Myr, respectively (Kadish et al. 2014) using (Hartmann 2005) isochrons. Kadish et al. (2014) separated the outer ridged unit of the Arsia FSD into three concentric regions and found a progressive increase in age with distance away from the Arsia edifice. The authors argued that such a relationship would be expected for a thick, slowly retreating ice sheet which shields the interior of the FSD from all but the largest impacts. Ideally, cosmogenic radionuclide measurements could provide surface exposure ages to document glacial recession (e.g., Staiger et al. 2006), but such data has not yet been acquired on Mars.

The ridged units in the Martian FSDs are assumed to be deposited during glacial recession based on their similarity to terrestrial analogs in the Dry Valleys of Antarctica (Head and Marchant 2003) and Iceland (Lucchita 1984; Williams 1978). Although the crater density measurements made by Kadish et al. (2014) appear to support this hypothesis, they do not rule out a scenario in which ridges are deposited during glacial advance. If moraines are deposited at the ice margin during advance, then subsequent burial of the ridge by the overriding ice sheet would shield the ridge from further impacts until obliquity changes gradually allowed the ice to sublimate back into the atmosphere. Thus, the crater density observations provide a surface exposure age estimate (time since ice sheet removal) and do not represent the age of features formed before or during ice sheet advance. Although the crater density measurements made by Kadish et al. (2014) help constrain the rate of ice margin retreat, they do not provide a test for determining whether ridge deposition happens during ice retreat or advance. Thus, additional observations and theoretical work needs to be done in order to test these hypotheses.

### Problems with current interpretation

The interpretation of the ridged unit as a series of cold-based glacier moraines deposited over a long duration (∼100 Myr) of retreat of the ice margin poses a conundrum regarding the Amazonian climate. In order to deposit a moraine, the ice margin needs to be roughly stationary while ice flow continues to advect ice and englacial/supraglacial debris to the ice margin. The location of the ice margin is a sensitive function of the aerial extent and rates of ablation and accumulation but is stationary when the total mass accumulated over the ice sheet is equal to the mass ablated (steady state). The conundrum arises from the observation that the moraines are so closely spaced (∼ 1 km) which accounts for a change of less than 1% of the length of the ice deposit assuming precipitation occurs on the slopes of the volcanic edifices as indicated in GCM results by Forget et al. (2006). Such small changes in the ice margin position would involve similarly small changes in the average ablation and/or accumulation rates over the ∼ 1 Myr (Kadish et al. 2014) timescale required to deposit an individual ridge. How did the glacial mass balance continuously change by such small amounts in order to form hundreds of ridges at Arsia (Kadish et al. 2014) when the dominant frequency of obliquity change happens over 120 kyr timescales (Schorghofer 2008; Ward 1992)?

Superposition of remnant ice deposits (smooth unit) on ridges (Fig. 1d, (Shean et al. 2005) and intersecting ridge sequences (Fig. 1c) provide geomorphic evidence for episodes of glacial advance following times of retreat within the Pavonis and Arsia FSDs. These features suggest several episodes of retreat and advance may have taken place (Shean et al. 2005) and also show that ridges can remain preserved even after being overrun by a later episode of ice sheet advance which may complicate interpretation of the ridge formation sequence.

### Potential ridge formation mechanisms

The dry, cold Amazonian climate of Mars limits the scope of potential ridge-forming mechanisms to those which would operate under cold-based, dry glacial conditions. Given the dominance of obliquity on the Amazonian climate (e.g., Tanaka 2004; Schorghofer 2008; Schorghofer 2006), we assume obliquity-driven climate change is primarily responsible for ridge formation and that the formation of a surface sublimation lag is the primary debris source. This lag acts to reduce the rate of ice ablation via sublimation. Given these assumptions, there are at least four potential processes for forming ridges:

1. During retreat of the ice margin, relatively warm periods (during times of low obliquity) may temporarily enhance ice flow and stall the migration of the ice margin to result in a ridge.
2. During glacial advance, the margin of the ice deposit may be slowed during times of high obliquity (cooler ice temperatures) which could allow even a low sublimation rate to maintain a temporarily fixed ice margin position—allowing debris to accumulate and a ridge to form.
3. Renewed ice accumulation at the head of a sublimating ice deposit during high obliquity may produce a kinematic (velocity) wave which may collect surface debris and deposit a ridge upon reaching the ice margin.
4. The steepness of the ice margin is expected to increase along with the ice viscosity during cold temperatures experienced during high obliquity at the equator which may cause slope failure of the surface lag and the formation of a ridge at the ice margin.

These processes may work in concert or independently to produce ridges of varying sizes depending on the dominant process at a given point in time. Furthermore, this list is not exhaustive but represents the most likely mechanisms based on our assumption of cold-based glacial conditions.

We investigate the process(es) responsible for depositing ridges by combining results from a thermomechanical ice sheet simulation with an analysis of the morphology of a series of ridges north of Pavonis Mons. Our model inputs assume ice accumulates in accord with GCM results at high obliquity and ice ablation occurs at a rate associated with the formation of a protective lag cover.

Understanding the process of ridge formation is an essential component to decoding the Amazonian climate change record contained in the FSDs and for understanding how active the Martian water cycle was in the recent past.

## Methods

### Stereo images and observations of moraine characteristics

**Fig. 2:**
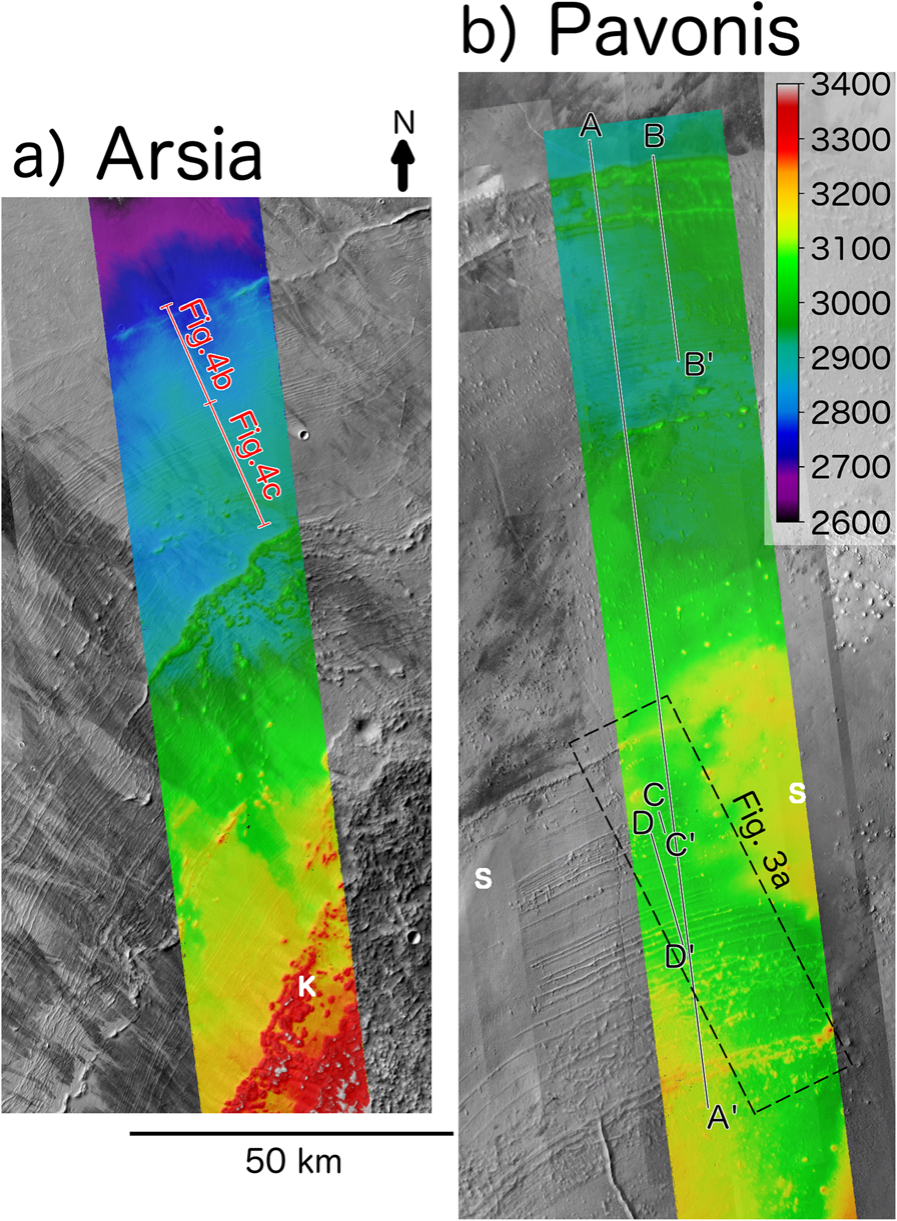
Topography and morphology of the distal portion of the Arsia (**a**) and Pavonis (**b**) FSDs. CTX imagery and transparent stereo topography (colored regions) shows ridge sequences of parallel ridges together with smooth (labeled with “S”) and knobby (labeled with “K”) units. The smooth unit (interpreted as remnant debris-covered ice by Shean et al. 2005) superposes ridges in the Pavonis FSD and indicates that the flow of ice responsible for forming the smooth unit was not erosive. Ridge spacing and cross-sectional area measurements using this data are shown in Figs. 3 and 4

**Fig. 3:**
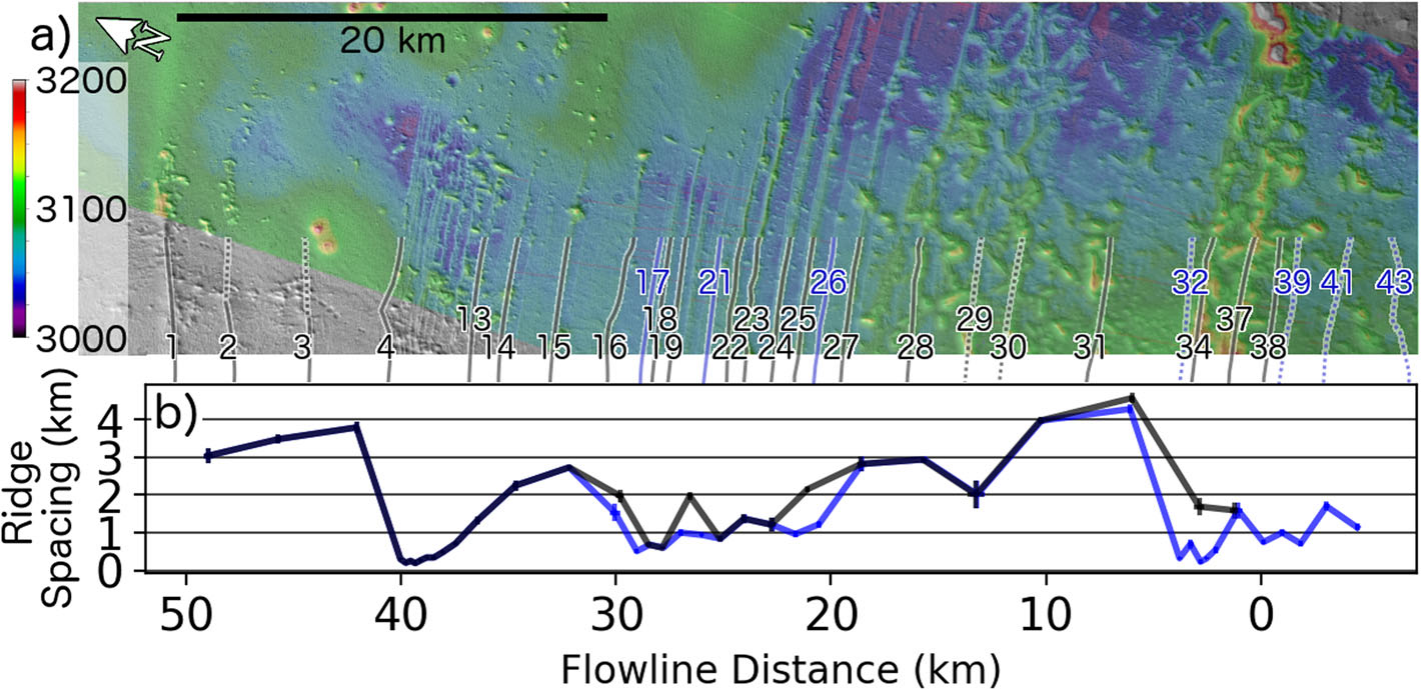
CTX imagery shown with transparent stereo topography of interior ridge sequence and calculated ridge spacing. **a** Section of CTX global mosaic (rotated to correspond to the x-axis of **b**) illustrating the emergence of parallel ridges from beneath the smooth unit (upper left) with black lines/numbers indicating ridges which are distinct in both CTX imagery and the stereo DEM (blue ridges are indistinct in one of these datasets). Dotted lines indicate poor ridge expression at this location, but, if black, has a more distinct section further to the east. **b** Mean spacing between individual ridge pairs (using three to five measurements per ridge pair) as a function of distance along model flowline (Fig. 1b) with error bars reflecting the standard deviation in the spacing between ridge pairs (generally smaller than the line width). The black line indicates spacing between high-confidence ridges only whereas blue line includes all ridges

In contrast with ridges in the Arsia FSD, the Pavonis Mons ridges are generally wider, taller, are exposed over a larger portion of the FSD, and exhibit a quasi-periodic pattern in their spacing which make them an intriguing feature to study (Figs. 2 and 3). A CTX stereo DEM (18 m/pixel resolution) was produced at the distal portion of the Arsia FSD (Fig. 2a) and the medial section of the Pavonis FSD (Fig. 2b) using the Ames Stereo Pipeline. DEM production made use of raw MOLA profiles as control points to improve quality (Beyer et al. 2018). The vertical precision of the DEMs are ≈ 2.46 m (see Hemmi and Miyamoto 2017 for a DEM generation workflow description).

The spacing between ∼ 40 ridges are measured using the CTX global mosaic (5.5 m/px) of the FSD interior (Fig. 3a). Depending on ridge distinctness, three to five measurements are obtained between each pair of ridges in a direction perpendicular to the ridge crests. A combination of CTX images and a stereo CTX DEM are used to identify ridges with varying degrees of confidence (Fig. 3b). In some cases, ridges are indistinct in the imagery but resolved in the DEM. High-confidence ridge identifications are prominent in both the imagery and the DEM (black numbers/lines in Fig. 3a, b), while lower-confidence identifications (blue numbers/lines in Fig. 3) are only expressed in one of the two datasets. Finally, the dotted lines in Fig. 3a indicate that the ridge is more prominent at a location outside the region shown.

The mean spacing between ridges varies in a quasi-periodic fashion in which ridges are densely spaced in the southernmost portion of the FSD, become more widely spaced to the north, with this pattern repeating again (Fig. 3b) before the ridges become extremely faint in the northern portion of the FSD. A few ridges near the FSD perimeter (along profile B-B′ in Fig. 2b) are very prominent. Error bars in Fig. 3b are associated with the standard deviation in the ridge spacing (3 to 5 measurements per ridge pair) and are generally smaller than the width of the plotted. The line graph in Fig. 3b is shown at the same horizontal scale as Fig. 3a such that the positions of points along the x-axis of Fig. 3b lie approximately halfway between ridges shown in Fig. 3a.

**Fig. 4:**
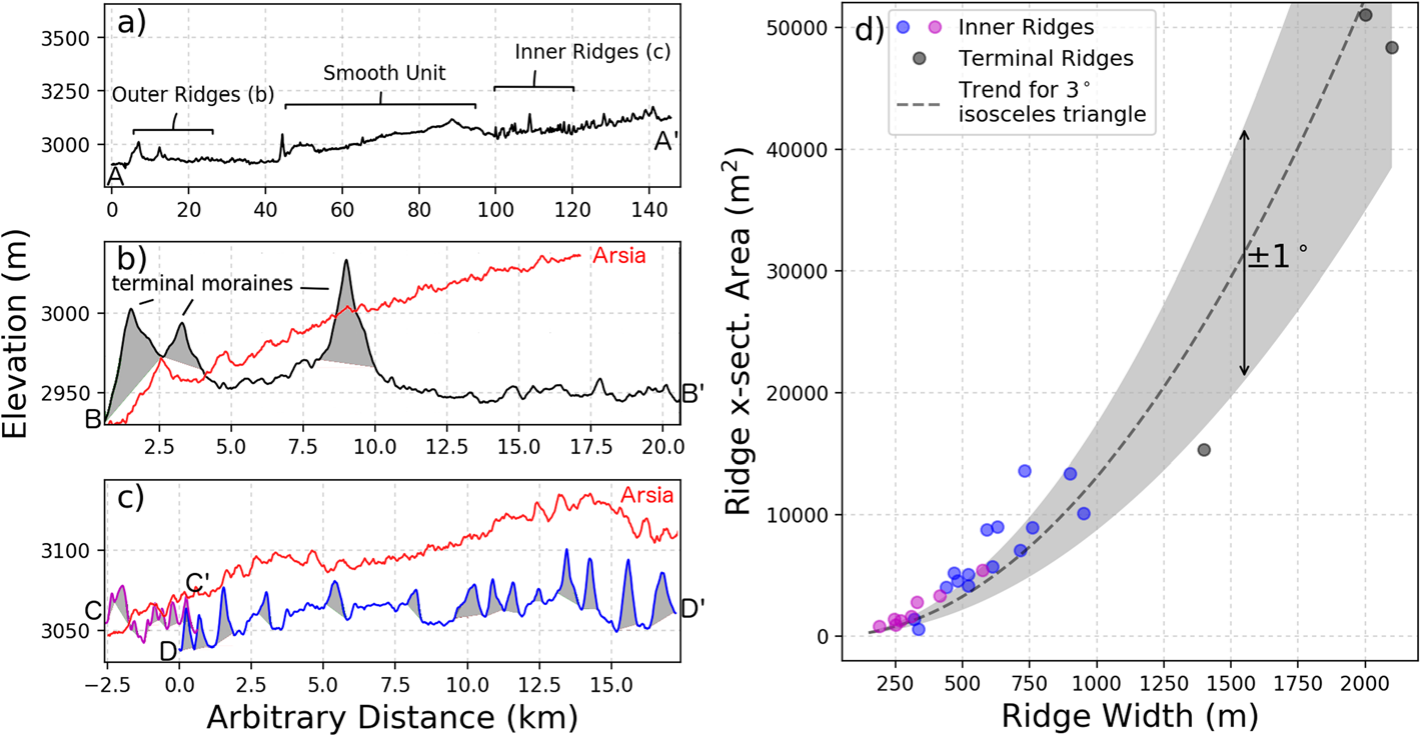
Ridge topographic profiles and cross-sectional areas measured from the CTX stereo DEM shown in Fig. 2. **a** This section of the FSD consists of outer ridge, smooth, and inner ridge units which rest on a regional slope of 0.1^°^. We, in agreement with prior work, interpret the outer ridges as terminal moraines (**b**) which are much larger than the interior ridges (**c**). The red lines in (**b**) and (**c**) indicate that ridges at Arsia are smaller and more closely spaced than at Pavonis (black, magenta, and blue lines). **d** The increase in cross-sectional area (y-axis) with ridge width (x-axis) generally follows the trend given by an isosceles triangle with flank slopes of 3^°^ (dashed line) although the smaller, inner ridges tend to have steeper slopes, but well below the angle of repose

The amount of material within the ridges provides an additional constraint on the deposition timescale and debris layer thickness (Kadish et al. 2014) as will be explored in the “Results and discussion” section. We extracted several topographic profiles (A-A′, B-B′, C-C′, and D-D′ in Fig. 2b) in order to determine the ridge cross-sectional area (shaded regions in Fig. 4a–c). The results are summarized in Table 1 for measurements of the 26 most prominent ridges which can be resolved by the CTX stereo DEM. The ridges found at the perimeter of individual flow units (identified using sets of parallel ridges) are much larger than the inner ridges (compare black line in Fig. 4b with magenta and blue lines in 4c) which are themselves larger than ridges found at Arsia Mons (red lines in Fig. 4b, c; Head and Marchant 2003). Individual ridge width and cross-sectional area measurements from each topographic profile are shown in Fig. 4d. The increase in cross-sectional area with ridge width generally follows that of an isosceles triangle with flank slopes of ∼ 3° (dashed line), although many of the smaller ridges have steeper flanks (Fig. 4d). This slope morphology contrasts with terrestrial washboard moraines, ribbed moraines, and “push ridges” which have heights of less than 5 m and exhibit steep (> 10^°^), asymmetric slopes in which the distal slope is steeper than the proximal (Price 1970).

**Table 1:** Pavonis ridge statistics

	Number	Ridge width	X-sectional area	Ridge spacing
Extracted from CTX stereo DEM ^a^				
All ridges	26	660 ± 480 m	9000 ± 12,500 m^2^	
Non-terminal ridges	23	500 ± 210 m	5200 ± 3800 m^2^	
Extracted from CTX image mosaic				
Prominent, non-terminal ridges	30			1.7 ± 1.3 km
Prominent + minor, non-terminal ridges	42			1.3 ± 1.1 km

^a^Generated from CTX images D08_030309_1850_XI_05N115W and D08_030375_1850_XI_05N115W

Both the ridge spacing (which constrains the ice margin velocity if a 120 kyr obliquity-driven formation timescale of ridge pairs is assumed) and cross-sectional area provide useful information which can be combined with the ice sheet simulation results.

### Numerical simulation

To investigate the influence of obliquity on glacier dynamics and to constrain the ice flow velocity, we performed numerical simulations by adapting the open-source Parallel Ice Sheet Model (PISM) (Winkelmann et al. 2011) to Martian conditions. PISM is a thermomechanical ice sheet and ice shelf model which accounts for the influence of a basal heat flux, strain heating, and climate forcings on the ice temperature. Although the model is capable of 3D simulations which incorporate basal sliding, we only require the shallow ice approximation component of the model which simulates deformation and flow of a grounded, cold-based, ice sheet (see, for example, Greve 1997; Marshall et al. 2000; Payne 1999). PISM solves the following pair of time-evolving partial differential equations describing mass and energy conservation:

1 $$ \frac{\partial H}{\mathrm{\partial t}}=M-\nabla \cdotp \overset{\rightharpoonup }{Q} $$

2 $$ \frac{\partial T}{\partial t}=\frac{k}{\rho {c}_p}\frac{\partial^2T}{{\partial z}^2}-\kern0.5em \overset{\rightharpoonup }{U}\cdotp \nabla T+\Sigma $$

where *H* is the ice thickness, *M* is the surface ice mass balance, and $\overset{\rightharpoonup }{Q}$ is the ice flux (vertical integral of the horizontal velocity). The change in temperature (*T*) with time (Eq. 2) is given by the vertical conduction, horizontal advection, and strain heating (Σ) terms (given from left to right) (see Bueler et al. 2007; Winkelmann et al. 2011 for details) where *k* is thermal conductivity, *ρ* = 910 kg m^− 3^ is the ice density, *c*_p_ = 2009 J kg^− 1^ K^− 1^ is the ice specific heat capacity, and $\overset{\rightharpoonup }{U}$ is the horizontal velocity.

To calculate the horizontal velocity at elevation (*z*) the simulation vertically integrates the shear strain rate given by Glen’s Law (*n* = 3, Glen (1955)) which gives:

3 $$ \overset{\rightharpoonup }{U}=-2{\left(\rho g\right)}^n\nabla {h}^{n-1}\left[{\int}_b^zA\left({T}^{\ast}\right)\left(h-\zeta \right) d\zeta \right]\nabla h $$

where *h* is the ice surface elevation and *A(T*^*∗*^*)* is a temperature-dependent ice softness parameter given by (Paterson and Budd 1982). Equations 1, 2, and 3 are solved on a 3-D rectangular grid with a horizontal spacing of 500 m and a vertical spacing that decreases from 170 to 27 m toward the base of the ice following a quadratic function (PISM authors 2014). The elevation of the base of the ice and the ice surface are given by b and h, respectively in Eq. 3.

Laboratory experiments by Goldsby and Kohlstedt (2001) and Durham et al. (2010) suggest that the value of *n* decreases at low differential stress (< 100 kPa) and is also dependent on ice grain size despite terrestrial field evidence for *n* = 3 behavior even at low differential stress (Budd and Jacka 1989; Cuffey and Paterson 2010). The flow law proposed by Goldsby and Kohlstedt (2001) generally results in faster ice flow rates than Glen’s Law at low stress which motivated (Pettit and Waddington 2003) to adopt an additional, *n* = 1, term to Glen’s Law in order to model ice deformation at low differential stress such as encountered near an ice divide. Although the lower gravity on Mars will tend to reduce the driving stress, we will be focusing on thick (∼ 2 km) ice sheets which generally result in differential stresses in excess of 100 kPa (see “Results and discussion” section) where *n* = 3 behavior tends to dominate (Parsons et al. 2011). However, use of Glen’s Law may underestimate ice flow velocities on Mars especially for thin or gently sloping ice deposits.

### Application of the PISM model to Mars

We are the first to apply PISM to Mars, and therefore adjusted a number of configurable parameters. We change the value for gravity to 3.728 m s^− 2^, disable basal sliding (cold-based glaciation), and apply a spatially and temporally constant basal heat flux of 30 mW m^− 2^ based on the estimated flux for Tharsis (Plesa et al. 2016), which is somewhat higher than the estimated global average of 20 mW m^− 2^. Thermal conductivity of the ice is allowed to change with temperature due to the colder Martian temperatures using the following relationship from Greve and Blatter (2009):

4 $$ k(T)=9.98{e}^{-0.0057T} $$

where *T* is the temperature in Kelvin. This equation results in *k* = 2.85 W m^− 1^ K^− 1^ at *T* = 220 K compared with a value of 2.1 W m^− 1^ K^− 1^ typical of terrestrial studies (PISM authors 2014) resulting in a factor of 1.36 faster rate of heat conduction within Martian ice deposits compared with terrestrial ice sheets.

The ice flow simulation was conducted on the 128 pixels per degree Mars Orbiter Laser Altimeter (MOLA) DEM regridded to 500 m resolution. In order to reduce computational demand associated with a high spatial resolution, we use PISM in a flowline mode where flow occurs along a single axis (x-direction) which follows the steepest gradient down the northwest flank of Pavonis Mons and through the FSD (white line in Fig. 1b). For the purposes of determining the flowline trajectory, we apply an averaging window (50 km in radius) to the MOLA topography. The steepest downslope direction on the smoothed terrain starting from an initial point near the summit of Pavonis defines the flowline location. Once the flowline is calculated, the 500 m/pixel resolution MOLA elevation values along the flow line are adopted as the basal topography. The smoothing avoids a meandering course or one that stops in small depressions to produce a downslope trajectory that would more closely resemble that of a thick ice sheet. Despite this smoothing, a broad depression within the FSD north of Pavonis Mons, interior to the ridged unit, causes the downslope profile to terminate. We forced the profile to continue on a northward trajectory in order to follow the central axis of the Pavonis FSD (Fig. 1b).

### Ice accumulation

**Fig. 5:**
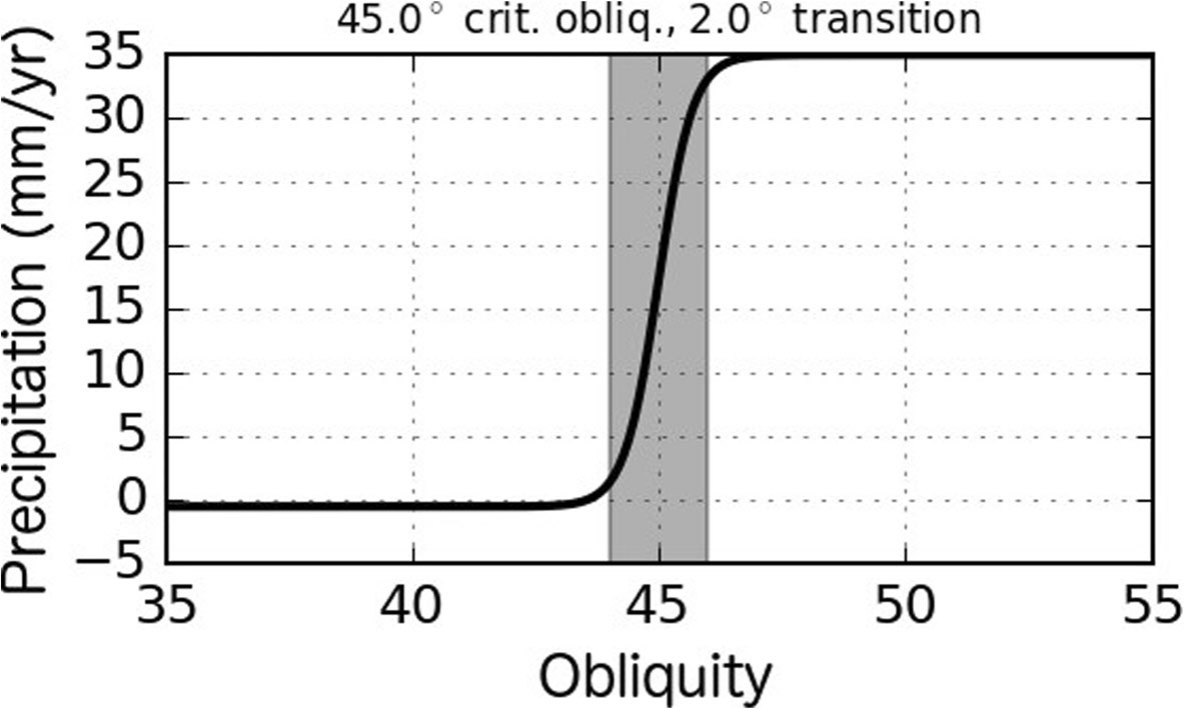
Obliquity-dependent precipitation rates assumed for the accumulation zone. The model assumes precipitation follows a logistic curve with the transition from ablation to precipitation (35 mm/year) occurring over a 2^°^ window in obliquity (shaded region) centered at 45^°^. The rate of ablation varies with temperature (dependent on obliquity and surface slope, see Figs. 8 and 9)

The model assumes an ice-free initial condition at an initial start time of *t* = − 26 Myr. As time progresses, ice accumulation occurs over a zone on the flank of Pavonis with basal topography between 5 and 12 km during times when obliquity approaches or exceeds 45^°^ based on high-obliquity GCM results from Forget et al. (2006). Figure 5 gives the relationship between ice accumulation (y-axis) and obliquity assumed by our model. The shape and amplitude of this logistic function was chosen after several iterations of simulating ice flow on Pavonis Mons in order to produce an ice deposit which remained within the extent of the FSD but extended beyond the set of interior ridges shown in Fig. 3a.

**Fig. 6:**
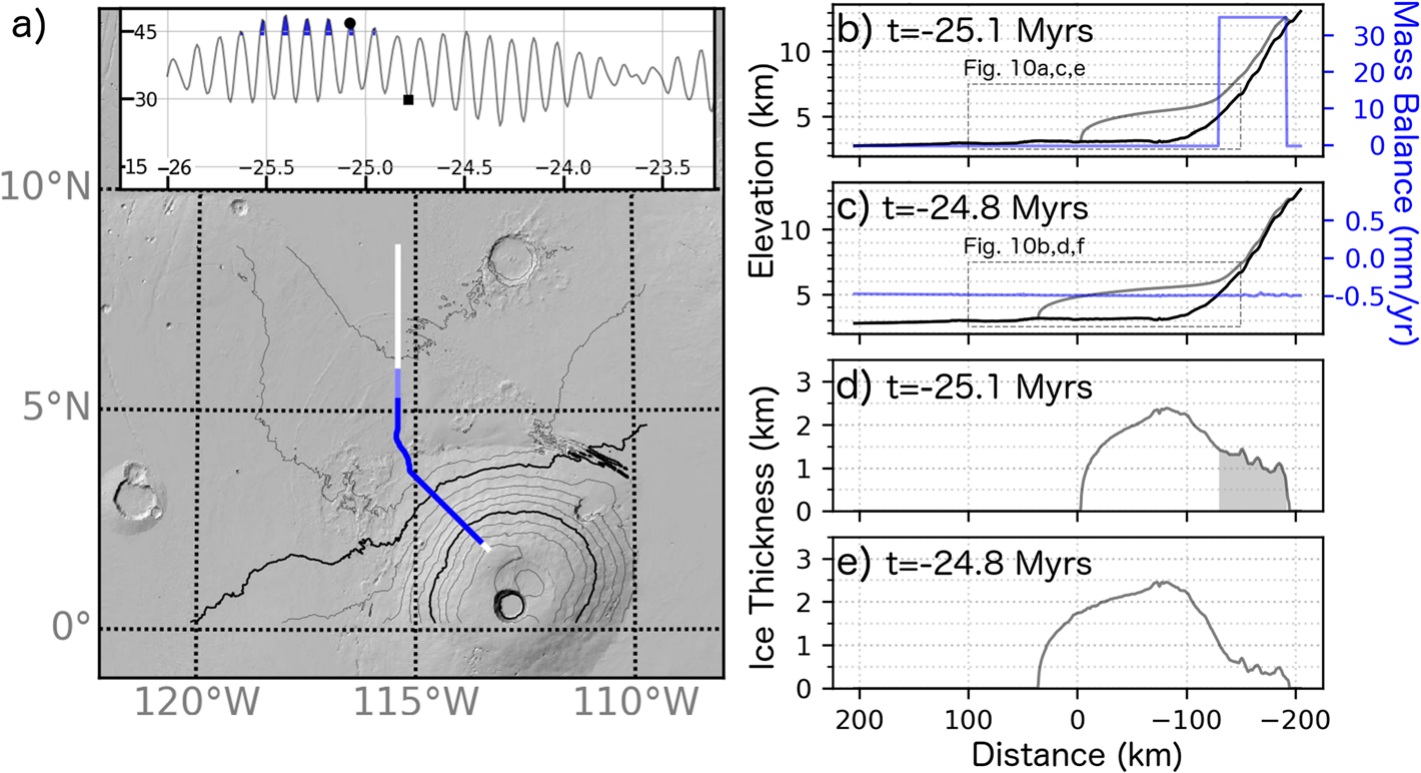
Model snapshots illustrating assumptions regarding the timing and location of ice accumulation. Inset plot shows obliquity history at the beginning of the simulations with time and obliquity on x and y axes, respectively. Precipitation occurs at times indicated by the filled blue regions (see Fig. 6). **a** MOLA hillshade contour (1 km interval) map indicating location of model flowline (white line) and extent of ice in the model (transparent blue lines) at − 25.1 (circle in inset) and − 24.8 Myr (square in inset). Topographic profiles of the ice deposit at − 25.1 and − 24.8 Myr are shown in **b** and **c**, respectively with the blue line (and right y-axis) indicating ice accumulation/ablation rate. Ice thickness for these times are shown in **d** and **e** with the shaded region in **d** indicating extent of ice accumulation

Snapshots of the model during high (47^o^ at *t* = − 25.1 Myr) and low (30^o^ at *t* = − 24.8 Myr) obliquity are shown in Fig. 6 (circle and square symbols, respectively, in inset) to illustrate our mass balance assumptions. The extent of the flowline model domain is given by the white line in Fig. 6a, with dark and light blue lines indicating the extent of ice at *t* = − 25.1 and − 24.8 Myr, respectively. Topographic profiles and instantaneous mass balance conditions at these times are given in panels b and c, while panels d and e give ice thickness profiles. The shaded region in Fig. 6d indicates the extent of the ice accumulation zone during high obliquity.

The GCM result from Forget et al. (2006) produces accumulation at Pavonis Mons at an approximate average accumulation rate of 35 mm/year (Earth year) at an obliquity of 45^°^ (compared with the blue line in Fig. 6b) assuming a water vapor source at the northern pole, although a south polar water source also results in accumulation at Pavonis. According to Forget et al. (2006), ice accumulation in the GCM was insensitive to obliquity at values larger than 40^°^, but precipitation may decrease once polar source regions become depleted over long durations of obliquity > 45^°^ when ice is unstable at the poles (Mischna et al. 2003). We assume that the accumulation zone of Pavonis Mons transitions from net ablation to net accumulation over an obliquity range of 2^°^ centered at 45^°^ following a logistic function (Fig. 5) under the assumption that precipitation is limited to times when polar ice becomes unstable. Moving the obliquity threshold for precipitation to lower values (by even a couple of degrees) resulted in many more ice accumulation events which causes ice to flow beyond the extent of the FSD.

### Ice ablation

The ablation rate is calculated assuming that the majority of ice loss occurs while ice is buried beneath a protective lag of 0.5 m thickness. Sublimation rates of exposed ice at mid-latitudes under current Mars climate conditions are estimated to be 4.1 mm/year (Earth years) based on repeat imagery (at 43.6^°^ N) of ice exposed by a recent impact (Dundas and Byrne 2010). Prior ice flow simulations for the Amazonian Tharsis ice sheets by Fastook et al. (2008) calculated sublimation rates associated with buoyancy- and turbulence-driven processes (Ingersoll 1970; Pathare and Paige 2005) which gives ≈ 5 mm/year net ablation rates at elevations just below zones of net accumulation. Although our calculated sublimation rate may be an underestimation of ice loss when glacial ice in the Tharsis region is newly exposed to low-obliquity climate conditions, the ice will become protected by a surface lag as dust entrained in the ice accumulates on the surface as a result of ice loss. Observational evidence for a reduced ice sublimation rate comes from the remnant ice protected by surface debris which makes up the smooth unit (Shean et al. 2005). Assuming that the maximum thickness of ice was less than ∼ 3 km (based on a perfectly plastic ice rheology (Shean et al. 2005)) and that the age of that ice must exceed the time since the most recent episode of high obliquity (> 5 Myr; Additional file 1: Figure S1b), then the average sublimation rate must have been less than 0.6 mm/year in order for the smooth unit ice to persist until today. This rough estimation is of similar magnitude to the ∼ 0.1 mm/year long-term (200 kyr) average ablation rate for massive ice buried below a 0.3 m active layer in the permafrost of Beacon Valley, Antarctica (Liu 2014).

In order to evaluate the timescale of the lag formation process and to determine the ice ablation rate in the model, we calculated the sublimation rate (*E*_s_) of buried, non-deforming ice using a water vapor diffusion equation modified from Chevrier et al. (2007):

5 $$ {E}_{\mathrm{s}}=\frac{D{M}_{H2O}{p}_{\mathrm{s}\mathrm{at}}}{LR{T}_s\rho}\left(1-\mathrm{RH}\right) $$

where *D* ≈ 5 × 10^4^ m^2^ s^− 1^ is the water vapor diffusivity through the lag which was measured for the JSC-1 Mars regolith analog (Hudson et al. 2007) at temperatures between 200 and 263 K and corresponding to a regolith pore size of 3 μm (Liu 2014). The molar mass of water is *M*_H2O_ = 0.018 kg/mol, *L* is the lag thickness in meters, *R* = 8.31 J/mol is the ideal gas constant, *T*_s_ is the ice surface temperature, *ρ* is the ice density, and RH is the atmospheric relative humidity. The water vapor saturation pressure (*p*_sat_) in exchange with ice is given by the Clausius-Clapeyron equation:

6 $$ {p}_{\mathrm{s}\mathrm{at}}={p}_t\exp \left[-\frac{H_{\mathrm{s}\mathrm{ubl}.}}{R}\left(\frac{1}{T_{\mathrm{s}}}-\frac{1}{T_{\mathrm{t}}}\right)\right] $$

where *p*_t_ = 611 Pa and *T*_t_ = 273 K give the triple point of water and *H*_subl._ = 5.1058 × 10^4^ J mol^− 1^ is the latent heat of sublimation. Combining Eqs.. 5 and 6 give the sublimation rate for a given ice surface temperature, burial depth, and atmospheric humidity. We can now associate an assumed lag thickness of 50 cm under dry (RH = 1%) atmospheric conditions with an ice surface temperature of 215 K (further addressed in Mars paleoclimate inputs section) to an ablation rate of 0.5 mm/year.

**Fig. 7:**
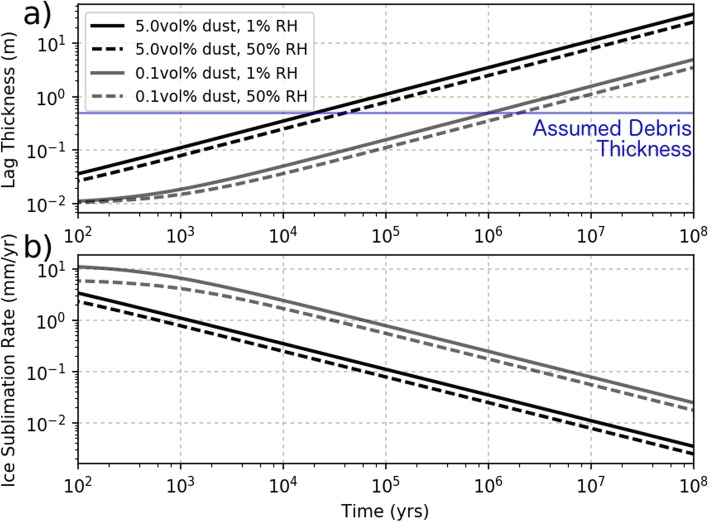
Time-evolution of lag thickness and ice sublimation rate of a Martian dirty ice deposit. **a** Modeled increase in lag thickness over time for different atmospheric humidities (solid vs. dashed lines) and ice deposit dust concentrations (black vs. gray lines) assuming an ice surface temperature of 210 K. The minor ticks on the x-axis give even increments from the base. The lag thickness in **a** is given by the buried ice sublimation rate calculated from Eq. 5 (**b**) integrated over time and multiplied by the dust concentration. This calculation assumed an initial debris thickness of 1 cm. The blue line in **a** gives the assumed debris thickness used to calculate the ablation rate in the simulation

Assuming an initial burial depth of 1 cm (the depth at which sublimation becomes diffusion-limited (Chevrier et al. 2008)), we calculated the lag deposit growth (Fig. 7a) and time-evolving sublimation rate (Fig. 7b) for a combination of RH = 1% or 50% and volumetric dust concentrations of 0.1% or 5% in the ice deposit for *T*_s_ = 210 K. The higher of these dust concentrations is associated with ice purity estimates from SHARAD radar observation of Gemina Lingula in the north polar layered deposit (Grima et al. 2009) while mid-latitude ice deposits have dust concentrations limited to < 10 vol% (Holt et al. 2008; Petersen et al. 2018; Plaut et al. 2009). Ice flow simulations used to constrain the ice rheology of a southern hemisphere mid-latitude ice deposit suggested dust concentrations of < 5 vol% (Parsons and Holt 2016).

The sublimation lag calculation shown in Fig. 7a indicates that a lag thickness of ≈ 50 cm would accumulate in 20 kyr or 1 Myr under dry atmospheric conditions with a dust concentration of 5% or 0.1%, respectively. The total ice lost at a given time in Fig. 7 can be calculated by dividing the lag thickness by the dust concentration. Therefore, the 50 cm thick surface lag assumed in our PISM simulation is associated with 10 m or 500 m of ice loss for dust concentrations of 5% and 0.1%, respectively.

**Fig. 8:**
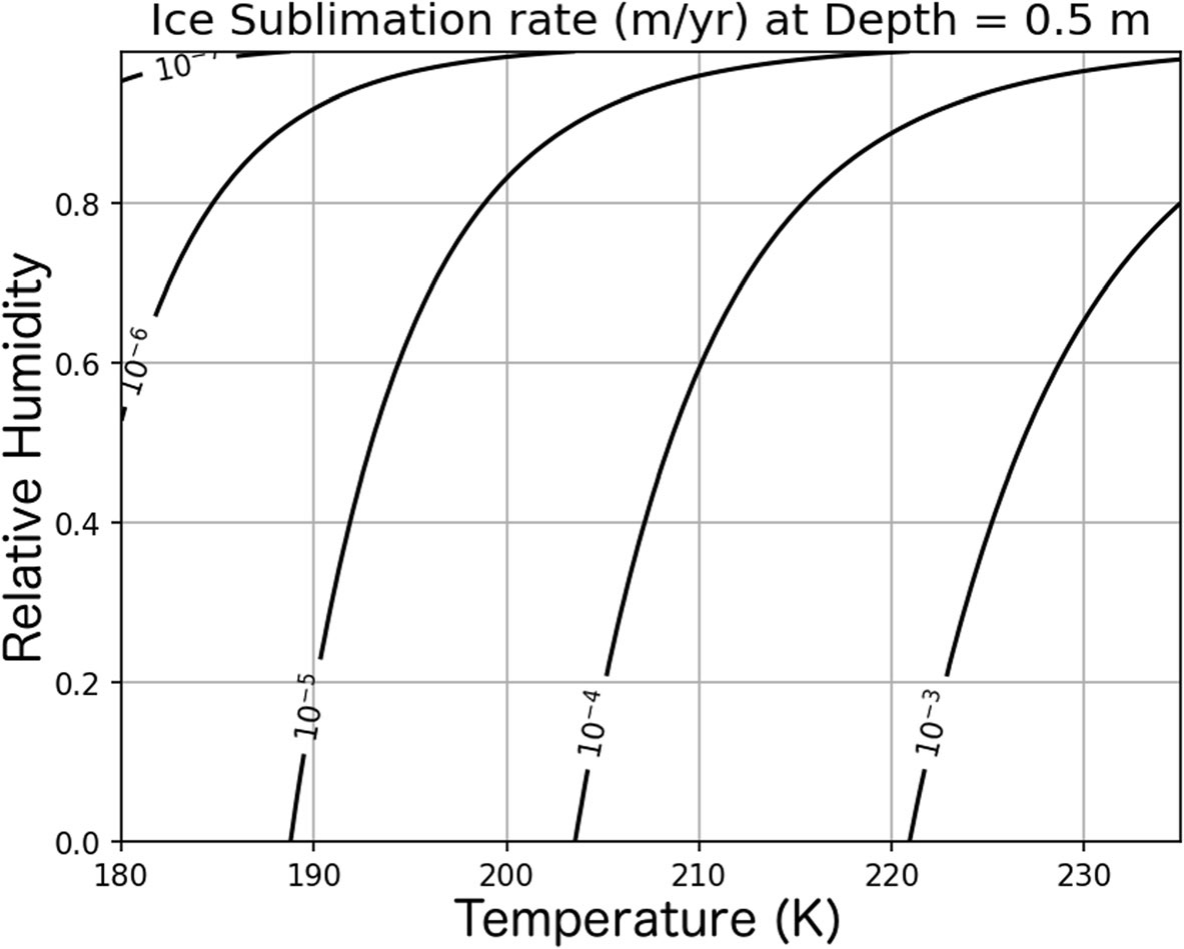
Dependence of ice ablation on temperature and relative humidity. Contours give sublimation rate for a given temperature (*x*-axis) and relative humidity (RH; *y*-axis) assuming an ice burial depth of 0.5 m based on Eq. 5. The model simulation assumes RH = 0.01 but allows temperature to vary with time and location along the flow line depending on obliquity and local slope, respectively

Assuming the presence of a 50 cm thick lag, Fig. 8 shows how the ice sublimation depends on the relative humidity and ice temperature. We allow the surface temperature to vary in the model (see below) which causes the sublimation rate to change with time, but the relative humidity is assumed to be dry RH = 1% at all times in the simulation.

The formation of the moraines themselves requires that debris be transported on or within the glacial ice in the absence of basal sliding. Even if the ice is initially exposed to the atmosphere, ice in the ablation zone will develop a sublimation lag due to the presence of englacial dust over a timescale that is short compared with the timescale of FSD formation. The 1 Myr timescale for lag formation with a dust fraction of 0.1 vol% and dry atmospheric conditions (RH = 1%) is short compared with the few million years it takes ice to flow from the accumulation zone to the inner ridges of the FSD (see “Results and discussion” section).

Ideally, we would hope to model the temporal and spatial variation in debris lag thickness within PISM as sublimation occurs and surface debris is advected (perhaps including dust deposition/erosion as additional parameters), but this computationally and work-intensive sub-model is left as a topic for future research. For the purposes of this study, our focus is to provide PISM with a set of conditions representative of the Martian climate and debris layer during FSD formation which can provide a basis for further investigation into the moraine deposition process. Our mass balance parameterizations were chosen after exploring several different maximum accumulation and ablation rates to give an ice extent that reaches the interior set of ridges but does not flow beyond the extent of the FSD during the last 26 Myr of known obliquity variations.

### Mars paleoclimate inputs

Constraining the age of the Pavonis FSD has been a difficult task as early crater counts using coarse (∼ 100 m/pixel) resolution THEMIS imagery failed to recognize that the thin, ridged unit of the FSD actually superpose fresh-looking impacts (Shean et al. 2005). Using CTX imagery, 49 craters which superpose the Pavonis FSD were counted by Kadish et al. (2014) to give an age estimate of 125 Myr. Mapping of the Pavonis edifice volcanic units (outside the FSD) using HRSC (15 m/pixel) resolution or better by Gwinner et al. (2011) suggests the most recent volcanic activity took place 80 ± 20 Myr ago and could have been contemporaneous with the presence of ice within the FSD (Shean et al. 2005). Given the uncertainty in both the timing of FSD formation and the Martian obliquity history, we rely on the most recent, well-constrained, obliquity history of Mars as a proxy for the past climate responsible for Pavonis glaciation and FSD formation.

**Fig. 9:**
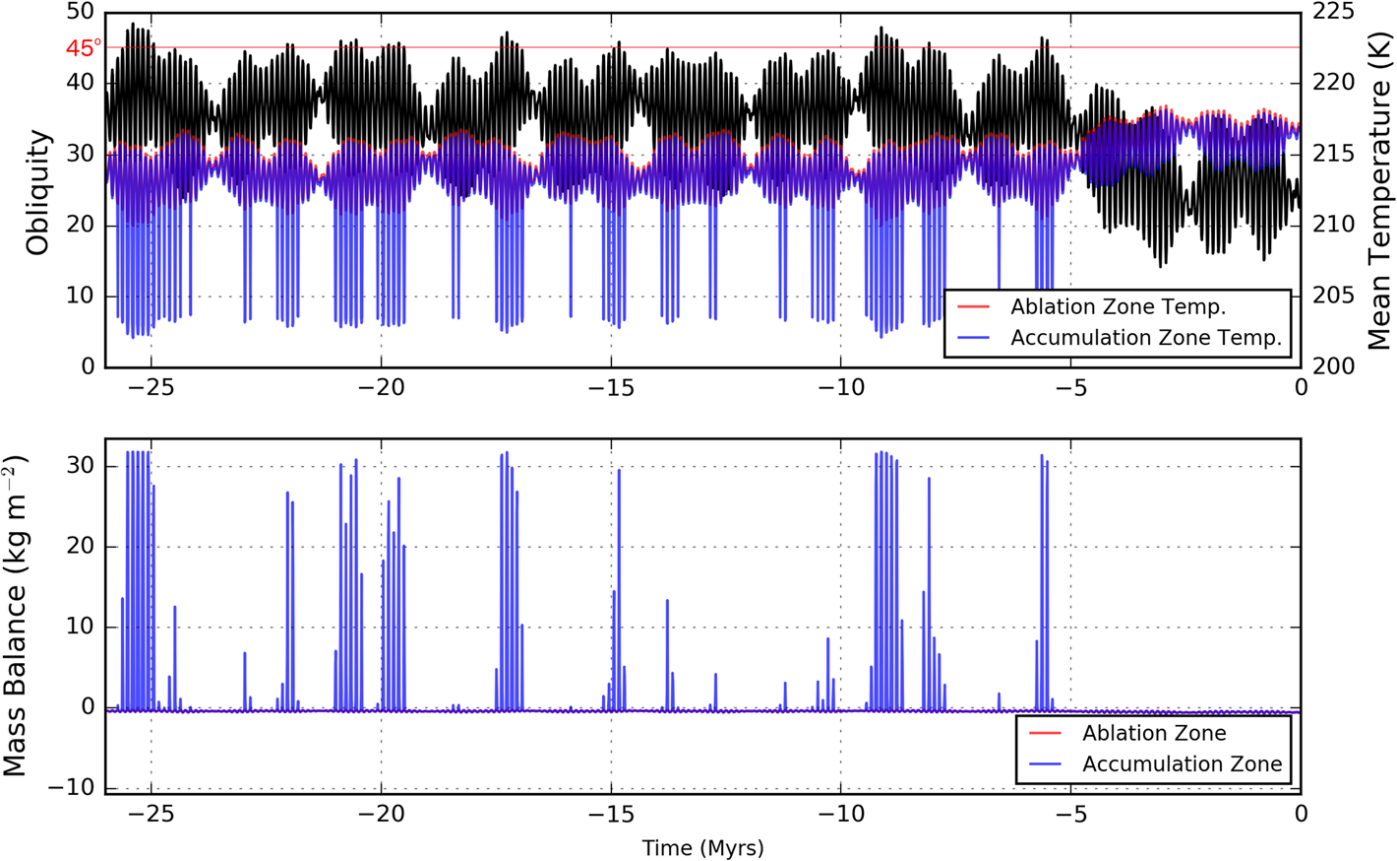
Obliquity and associated temperature and mass balance model inputs for the past 26 Myr. **a** Obliquity (black line) determined by Laskar et al. (2004) and the average surface temperature in the accumulation (red line) and ablation zones (blue line). If ice is accumulating in the specified accumulation zone, the surface albedo in the zone is changed from 0.3 to 0.4 resulting in a ~ 10 K drop in temperature. **b** The ablation rate changes with local temperature (Eq. 5), and ablation occurs everywhere at obliquity less than roughly 44^°^. The rate of accumulation depends on obliquity using the assumed relationship shown in Fig. 5

The obliquity of Mars over the past 26 Myr derived by (Laskar et al. 2004) (black line in Fig. 9a) was used as an input to our model in order to determine both the surface temperature and ice mass balance. We use this input as a proxy for variations experienced earlier in the Martian past and do not claim that FSD formation took place within the last 26 Myr. Prior to 26 Myr, the astronomical calculations used to determine obliquity begin to diverge when slight changes are made to spin-axis precession rate (Laskar and Robutel 1993; Additional file 1: Figure S1). Rather than evaluate various obliquity scenarios (which has already been done by Fastook et al. 2008 to match ice flow simulations to the maximum FSD extent), our aim is to simulate the influence of short-term climate variability on ice flow dynamics in order to investigate the process by which moraines are deposited. Using the obliquity-accumulation parameterization shown in Fig. 5 for the accumulation zone and an ablation rate given by Eq. 5 with *L* = 0.5 m elsewhere, PISM has the necessary mass balance inputs. Next, a connection needs to be made between obliquity and the surface temperature of the Pavonis ice sheet.

The Mars-year average insolation ($\overline{Q}$) at latitude (*ϕ*) is given by Ward (1974) which we have modified to approximate the influence of topographic slope:

7 $$ \overline{Q}=\frac{S}{2\pi \sqrt{1-{e}^2}}{\int}_0^{2\pi}\sqrt{1-{\left[\sin \left(\phi +\delta \right)\cos \theta -\cos \left(\phi +\delta \right)\sin \psi \right]}^2} d\psi $$

where *S* = 588.6 W m^− 2^ is the solar constant for the Martian orbital semi-major axis, *e* is orbital eccentricity, *θ* is obliquity, and *ψ* is an integration variable. The north-south component of the local slope is given by *δ* with positive values associated with pole-facing slopes. Adjusting the latitude by the local slope is a reasonable first-order approximation for slopes < 30^°^ based on full radiative balance calculations by Aharonson and Schorghofer (2006)) although it tends to slightly overestimate the influence of slope on temperature. We implement this slope-adjusted calculation in order to avoid more demanding computation that would be needed to produce temperature estimates at the ∼ 500 m topographic resolution of interest in this work.

Assuming radiative balance, $\overline{Q}$ is then used to determine the equilibrium surface temperature (*T*_eq_):

8 $$ {T}_{e\mathrm{q}}={\left(\frac{\left(1-A\right)\overline{Q}}{\sigma}\right)}^{1/4} $$

where *A* is surface albedo and σ is the Stefan-Boltzmann constant. Variation in obliquity and eccentricity over the past 26 Myr determined by Laskar et al. (2004) are used as inputs into Eq. 7. Although Mars has an average surface albedo of about 0.25, we use a value of 0.3 associated with the dust-covered Tharsis region (Christensen et al. 2001) for locations in the model experiencing ablation. For locations where ice is accumulating we use an albedo of 0.4 associated with values for the northern residual polar cap (Kieffer et al. 1976) as done in the previous Mars ice sheet models (Fastook et al. 2008).

An important note about Eq. 7 is that it is independent of the cyclic, ∼ 50 kyr variation in the argument of perihelion even though the instantaneous incoming solar flux at a given latitude is affected by this variation. However, Schorghofer (2008) used an alternative temperature calculation (the “standard thermal model”) to show that the obliquity signal dominates over the influence of the argument of perihelion at latitudes outside of ≈ 50–70^°^N and S.

Figure 9a shows how the average temperature of the ablation (red line) and accumulation (blue line) zones change as a function of time due to changes in obliquity (black line) and eccentricity. At equatorial latitudes, temperatures decrease during high obliquity while the opposite is true at the poles (e.g., Schorghofer 2008). The ∼ 5^o^ pole-facing slope in the accumulation zone results in colder temperatures than the ablation zone despite the ablation zone being at slightly higher latitudes. During times of high obliquity, the temperature in the accumulation zone is significantly reduced due to the increase in albedo assumed to be associated with ice accumulation. The surface temperature inputs to PISM vary with time according to the obliquity changes as shown for the “P000” scenario in Additional file 1: Figure S1b, but the surface slope changes induced by the accumulation and flow of ice are not updated within PISM during the simulation. Although the MOLA-based temperatures will closely match those at the thick, interior portions of the ice sheet, these prescribed surface temperatures will over-estimate the temperature at the steeper, pole-facing margin of the ice sheet (see “Results and discussion” section).

The computed ice mass balance model input is shown in Fig. 9b where obliquities between 44 and 46° produce accumulation rates smaller than the 32.4 kg m^− 2^ maximum (equivalent to 35 mm/year) based on the shape of the logistic curve shown in Fig. 5.

### Post-processing of PISM outputs

In order to determine when and where moraine deposition would be most likely from the PISM results, we saved ice surface velocities from the model every 2 kyr during the first 4 Myr of our simulation (which starts at − 26 Myr). We then imported these data into a Python script which tracked the advection of particles placed on the surface of the ice sheet using a 2D (time and space) linear interpolation.

The location of particle deposition is at the base of Pavonis with all particles being placed at the − 100 km point on the flowline (Fig. 1b) after ice advances past this point. After the first particle is advected 50 m toward the toe, a new particle is placed at the − 100 km location in a repeating process which mimics the development of a uniform-thickness sublimation lag. Once a particle is advected within 1 km of the toe of the ice sheet, it is assumed to be deposited and its flowline location is recorded.

## Results and discussion

**Fig. 10:**
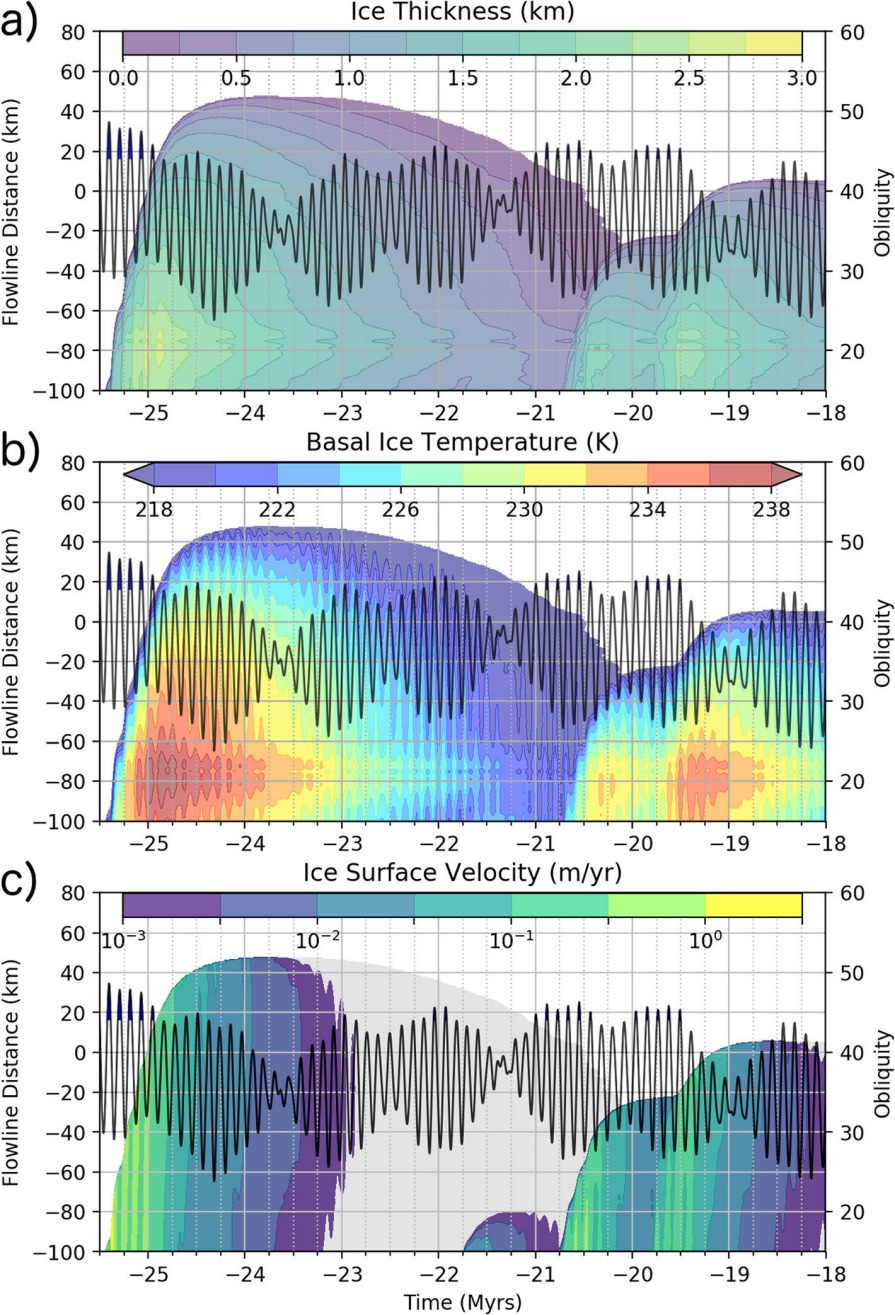
Model results depicting changes in the ice extent and ice deposit properties in the distal portion of the simulated ice deposit. Ice thickness (**a**), basal ice temperature (**b**), and ice surface flow velocity (**c**) are indicated by the colored contours plotted against the distance along the flowline shown in Fig. 6 (left *y*-axis) and time (*x*-axis). White regions in these panels contain no ice. The gray region in (**c**) indicates that ice is present, but the surface flow velocity less than 10^− 5^ m/year. The black line in each panel gives the obliquity history over this time period according to the right *y*-axis with blue filled sections indicating times of ice accumulation in headward section of the simulated flowline

Given our surface mass balance and temperature inputs (Fig. 9), PISM calculates the flow velocities within the ice sheet while accounting for the conduction of heat in the vertical direction, advection of heat in the horizontal direction, and internal generation of heat from deformation. The simulation produces an ice sheet of varying extent and thickness (Fig. 10a), but always stays within the boundaries of the FSD.

**Fig. 11:**
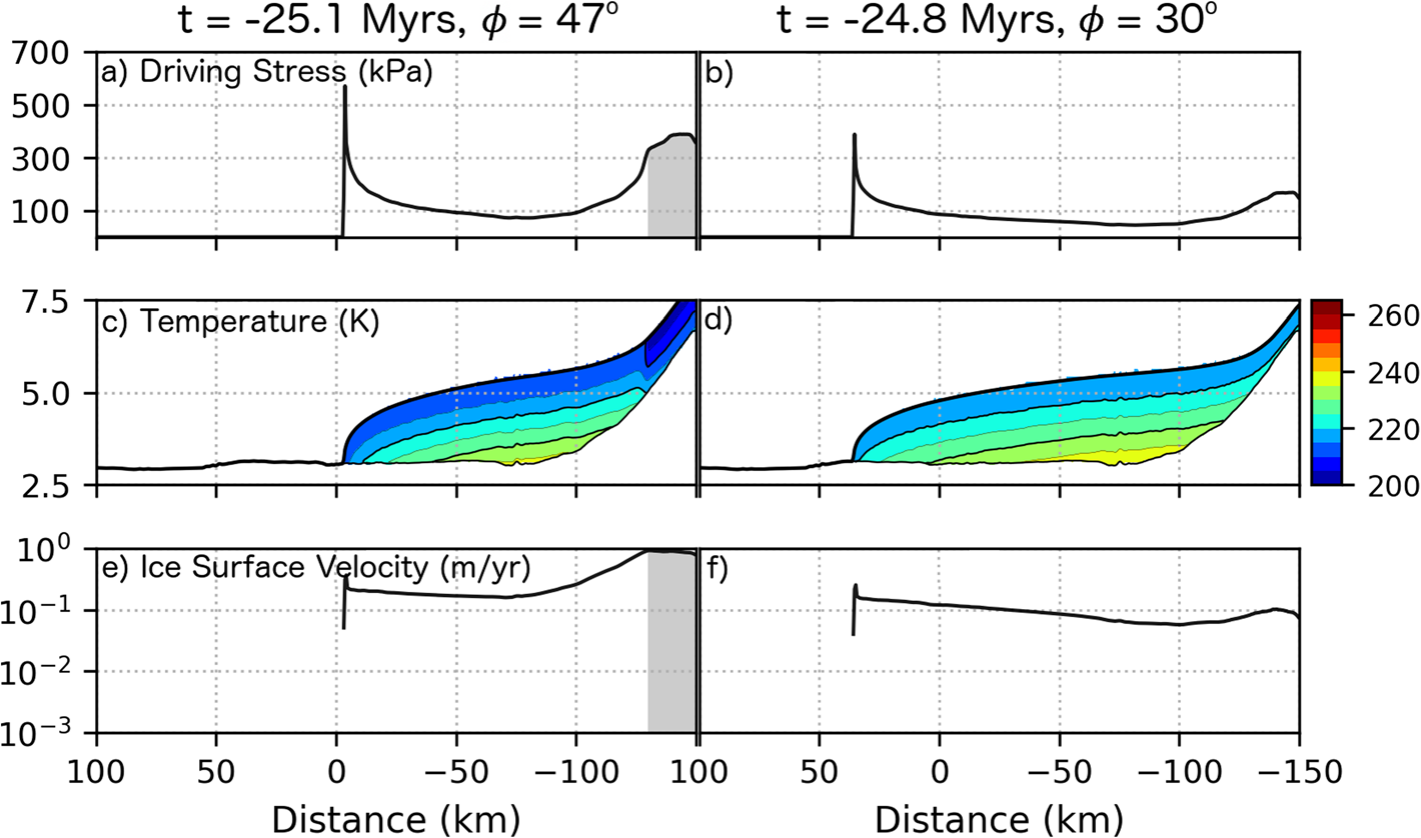
Simulation results depicting the ice deposit properties and flow velocities at high (47^°^) and low (30^°^) obliquities during ice sheet advance. Panels (**a**, **c**, and **e** give the basal driving stress, temperature, and ice surface velocity, respectively, at high obliquity (− 25.1 Myr) as shown in Fig. 6 a and b. Panels **b**, **c**, and **f** give the same set of properties at low obliquity (− 24.8 Myr)

Snapshots of the simulation at 25.1 and 24.8 Myr ago are provided in Figs. 6 and 11 (see Additional files 2, 3, 4 for animated plots of the simulation). The location of the model flowline is provided by Fig. 6a. The magnitude of the driving stress, ice temperature, and ice surface velocity are shown in Fig. 11 at these two times. Surface ice temperatures depressed by the increase in albedo during times of ice accumulation (right side of Fig. 11c) are conducted to the base of the ice sheet which reduces the ice flow rate and requires more ice accumulate (resulting in elevated stress, Fig. 11a) in order to drive ice flow. In the ablation zone, an ice thickness of 2.5 km (Fig. 6e) results in a surface to base ice temperatures difference of ≈25 K associated with the conduction of the basal heat flux (Fig. 11d). However, the temperature gradient within the ice fluctuates by up to 25% due to the propagation of surface temperature variations. Martian regolith thermal modeling by Grott et al. (2007) of 100 kyr periodicity climate variations with amplitude 10 K give a 10% variation in the Martian surface heat flux (proportional to the temperature gradient) albeit for a regolith with a lower thermal conductivity than ice. Heat produced from ice deformation is negligible—less than 0.03 mW m^− 3^ at the base of the ice sheet during/after accumulation events when deformation rates are highest.

Figure 10 provides a visualization of the changes in conditions at the distal portion (a flowline distance of − 100 km is associated with the base of Pavonis Mons) of the ice sheet over the first 7.5 Myr of the simulation. The ice thickness increases rapidly during episodes of accumulation and slowly thins during prolonged periods of ablation (Fig. 10a). There are changes to the basal ice temperature of less than 2 K in amplitude (Fig. 10b) caused by obliquity-driven temperature variations propagating through the ice deposit. The ice margin velocity peaks at between 0.1 to 1 m/year during brief periods (Fig. 10c) of ice accumulation but exponentially decreases with time after accumulation ceases due to a reduction in the driving stress as the ice sheet spreads out. Obliquity-driven temperature fluctuations perturb the ice flow slightly during glacial advance (e.g., at − 24 Myr). About 2 Myr after the last pulse of ice accumulation, the ice flow rate is less than a millimeter per year (gray zone in Fig. 10c) and the ice margin begins to retreat as the entire ice sheet continues to sublimate.

### Surface velocity response to temperature variations

**Fig. 12:**
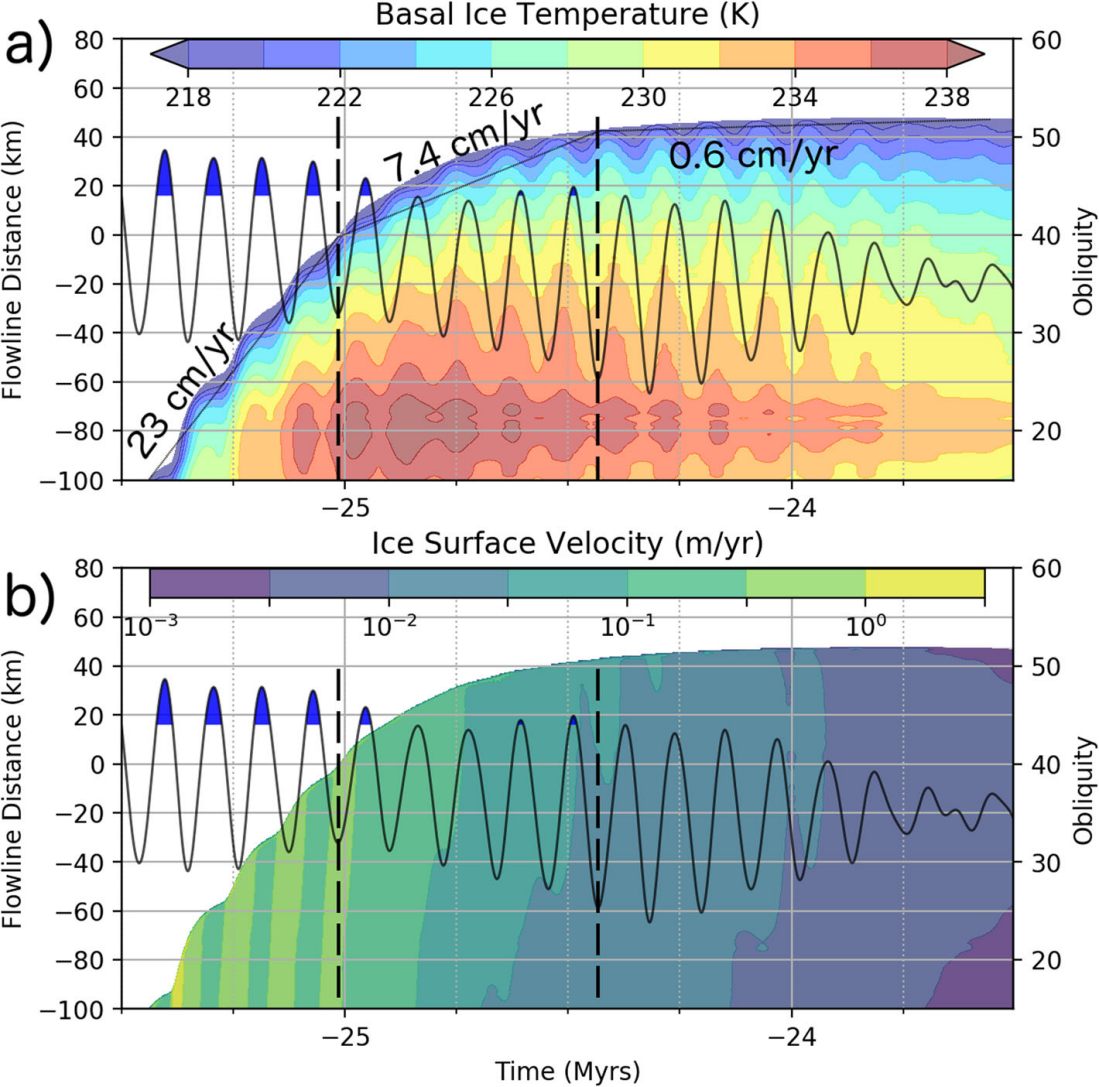
Increased time resolution model output showing details of the change in ice flow velocity in response to ice accumulation and obliquity-driven temperature change. Highest ice flow velocities follow episodes of ice accumulation. **a** Basal ice temperature increases with ice thickness (due to thermal conduction of the geothermal flux), while variations over a shorter timescale result from top-down conduction of obliquity variations. **b** Surface ice velocity response to episodes of accumulation (blue obliquity peaks) and obliquity-driven temperature changes. Dashed line indicate direction of velocity wave propagation (toeward after accumulation events, e.g., near − 25 Myr, and possibly headward in response to obliquity-driven temperature change, e.g., at − 24.4 Myr)

When obliquity is at a local minimum, the surface temperature reaches a local maximum. Temperature variations at the base of the ice sheet have a lower amplitude and are out of phase compared with the surface temperature due to dampening of the propagating, periodic, obliquity-driven thermal wave (e.g., Grott et al. 2007). The thinner ice near the toe of the glacier means that the downward propagation of the thermal wave reaches the base of the ice sooner at the toe compared with headward portions of the ice sheet. The right-most dashed vertical line in Fig. 12a helps to illustrate this effect. In this figure the obliquity signal is given by the black line (right y-axis) with the blue filled portions indicating times of accumulation at flowline distances less than − 130 km. The dashed line indicates the point in time associated with an obliquity minimum. The wave-like pattern in the temperature reaches a local maximum at the toe sooner (closer to the dashed line) than does the headward portion of the ice sheet.

The flow response to this temperature fluctuation causes the toe of the ice sheet to advance ahead of the headward portion of the ice sheet, and thin in the process. In this case, the increase in velocity is originating at the toe of the ice sheet and propagating in the headward direction as the low-obliquity temperature perturbation propagates downward (indicated by the slight rightward skew in the local velocity maximum relative to the dashed line in Fig. 12b).

### Surface velocity response to episodes of ice accumulation

During the series of ice accumulation episodes occurring during local maxima in obliquity between − 25.6 and − 24.9 Myr, accumulation of ice results in a surface velocity wave propagating from the headward portion of the ice sheet toward the toe. Once this velocity wave reaches the toe of the ice sheet, advance occurs rapidly (Fig. 12b).

### Particle advection calculation

**Fig. 13:**
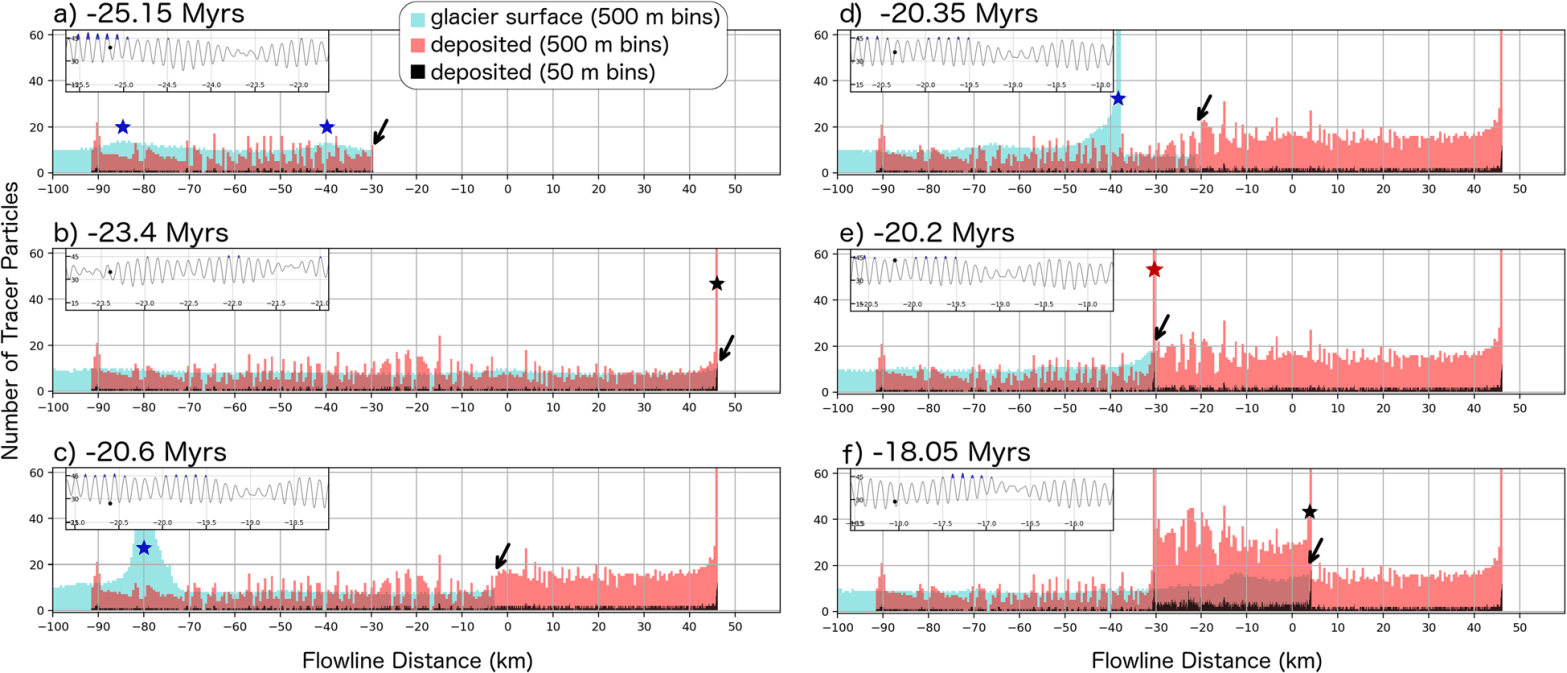
Ridge formation modeled by advection of tracer particles placed on the simulated ice sheet. Blue and red histograms give the number of tracer particles on the ice sheet surface and deposited on the substrate, respectively using a 500 m bin size (resolution of PISM simulations). Particles are individually placed at the − 100 km flow line position and are advected by the flowing ice. Once a particle has been transported 50 m, a new particle is placed at the − 100 km position. The black histogram gives the number of deposited particles using a 50 m bin size and represents the same data in the red histogram.

Computational limitations on the number of advecting particles we can track (spaced every 50 m) and the number of model outputs used (every 2 kyr) pose a challenge to directly simulate ridge formation due to the generation of large file sizes and long computation times. Although temporal interpolation between outputs and spatial interpolation between model grid points is necessary, the particle-tracking analysis shown in Fig. 13 provides insight into the potential ridge deposition mechanism.

Plots 13a–f show the density of particles on the ice surface and those that have been deposited on the substrate as blue and red histograms (500 m bin sizes), respectively. Between − 25.15(Fig. 13a) to − 18.05 Myr (Fig. 13f) the simulated ice sheet advances, retreats, and advances again. The location of ice margin is indicated by the black arrow in each plot. The blue stars indicate debris accumulations on the glacier surface which, depending on their size, may result in a ridge once they reach the ice margin. Ridge deposition events associated with a stationary ice margin are indicated with black stars whereas ridge deposition associated with a kinematic wave generated from an episode of ice deposition is shown with the red star. The black histogram boxes give the deposited particle concentration using a 50 m bins size but use the same data depicted by the red histogram.

A consistency check on the kinematic wave deposition hypothesis comes from estimating the longitudinal distance (Dist) along the ice deposit which surface lag debris of thickness (*L*) must be collected in order to deposit a ridge with a given cross-sectional area (*A*). Assuming that the ice margin is stagnant, and that both the lag layer and ridges are ice-free, this “debris collection distance” is:

$$ \mathrm{Dist}=A/L. $$

Using the lag thickness assumed in our model (*L* = 0.5 m) and a mean interior ridge area of 5000 m^2^ (Table 1) gives a distance of 10 km. By comparison, the mean width of the interior ridges is 500 m (Table 1). Therefore, regardless of how much time it takes to deposit a ridge, the ice flow velocity roughly 10 km from the ice margin must be 20 times the velocity of ice margin itself in order to deposit the required volume of material in the space occupied by the ridge. It is also worth noting, that this discrepancy between the ice margin velocity and ice flow velocity near the toe must subsequently disappear in order for ridge deposition to cease. This would happen either by the ice margin velocity increasing (as would happen during ice sheet advance) or by the ice flow velocity decreasing (which would promote retreat). The latter case represents the formation of terminal moraines, but the former case better explains the pattern of interior ridge spacing resulting from repeating episodes of ice deposition causing kinematic waves during ice sheet advance.

The changes to the margin of the simulated Pavonis ice deposit using our paleo-climate assumptions provide several insights into the deposition of ridges in the FSD. Our model results suggest that ridge formation is a transient process caused by surface velocity responses to quasi-periodic (120 kyr) obliquity changes. This result is a departure from prior interpretations which suggested ridges were the result of longer term (~Myr) periods of near steady-state conditions. We revisit the potential ridge-forming mechanisms described in the introduction in the context of our simulation results:

#### Conditions during ice margin retreat

Ice sheet thinning results in the cessation of ice flow during ice margin retreat even during times of low obliquity (warm ice conditions) (Fig. 10b, c). Therefore, advection of surface debris has halted during ice margin retreat and deposition of the surface lag can only occur passively as sublimation removes ice from below the thin debris layer (as depicted in Fig. 13c). Therefore, using the low sublimation rate and obliquity-driven accumulation assumptions in this work (Figs. 5, 8, 9), we find it very unlikely that FSD ridges were formed during glacial retreat.

#### Temperature variations during ice margin advance

Ice accumulation and, to a lesser extent, basal temperature changes affect the ice flow velocity in our simulations. During advance, the average ice sheet margin velocity is initially ~ 10 cm/year but slows to about 0.5 cm/year after a million years of flow (Fig. 12). Ice surface temperatures vary due to obliquity changes, which, after being conducted to the base of the ice sheet, produce temperature changes of less than 2 °C (Fig 10 and 12). These subtle temperature changes have little effect on the ice flow dynamics (Fig. 12b) and cannot produce significant kinematic waves that would perturb the surface debris layer. However, because our surface temperature calculation is based on basal topography and is not updated to reflect the topography of the ice deposit, the simulation may be underestimating the role of temperature changes on the ice flow dynamics. Even more important is the consequence that this assumption has on the ablation rate (discussed below) which is highly temperature dependent. Therefore, we cannot rule out the potential for temperature variations to cause variations in the ablation rate which would lead to the periodic deposition of moraines. Our simulations show, however, that such a process would need to happen during glacial advance in order for ice flow to advect debris to the ice margin which would be held in place during times of high ablation.

#### Renewed accumulation

A pulsating advance of the ice margin results from several episodes of accumulation during short-lived periods of high obliquity associated with the 120 kyr periodicity in obliquity (Fig. 12). Discrete episodes of accumulation generate kinematic waves in the ice deposit which produce thickness variations in the advecting debris layer (blue stars in Fig. 13a). However, during the initial advance at − 26 Myr, the amplitude of these accumulation-driven velocity perturbations is not significant enough to result in narrow zones of high debris thickness that would result in the formation of a ridge upon deposition at the margin. The − 100 km particle deposition location is likely responsible for this result because the fluctuation in ice velocity is much greater in the accumulation zone (and would promote greater variation in debris thickness) than at the base of Pavonis. However, in order to provide time for a surface lag to develop, we assume a particle deposition location which is about 30 km downslope from the edge of the high-obliquity accumulation zone.

Despite this assumption, a thick, narrow pulse of surface debris does form at about − 20 Myr when a stagnant toe is reactivated by renewed accumulation producing a propagating velocity “step” (Fig. 10c). In our simple particle advection analysis, this step in velocity causes particles to “pile-up” in front of the high-amplitude kinematic wave (blue star in Fig. 13d) and results in a local deposition of surface particles nearly 20 times more concentrated than that given by the assumed surface particle spacing of 50 m (black bars below arrow in Fig. 13e). The formation of this ridge is contingent upon the assumption that the re-activated ice sheet does not override the stagnant ice, but “flows into” the distal deposit in a way that advects the surface debris in process similar to a bulldozer.

#### Surface debris slope failure at ice margin

The steepness of the margin of flowing ice sheets on Mars is expected to be greater than their terrestrial counterparts due to reduced gravity. This conclusion can be reached by analyzing the shear stress which, at the base of the ice sheet of thickness (*H*)—given by:

9 $$ \tau =\uprho gH\frac{\partial h}{\partial x} $$

for the 2-D, flowline case. In order for Martian ice sheets to achieve comparable basal shear stresses to terrestrial ice sheets, Eq. 9 shows that the ice surface slope (∂*h*/∂*x*) must be greater on Mars to compensate for the reduction in *g*. Identical simulations performed under Earth gravity resulted in a decrease in the ice margin slope from roughly 15^°^ under Martian gravity to 10^°^ (see Additional files 1, 2, 3, 4).

However, the simulated ice margin slopes are still less than the angle of repose for unconsolidated dust on Mars of 28–35^°^ (Atwood-Stone and McEwen 2011) and the lateral continuity of FSD ridges suggest slope failure of surface debris is not the primary process responsible for ridge formation.

**Fig. 14:**
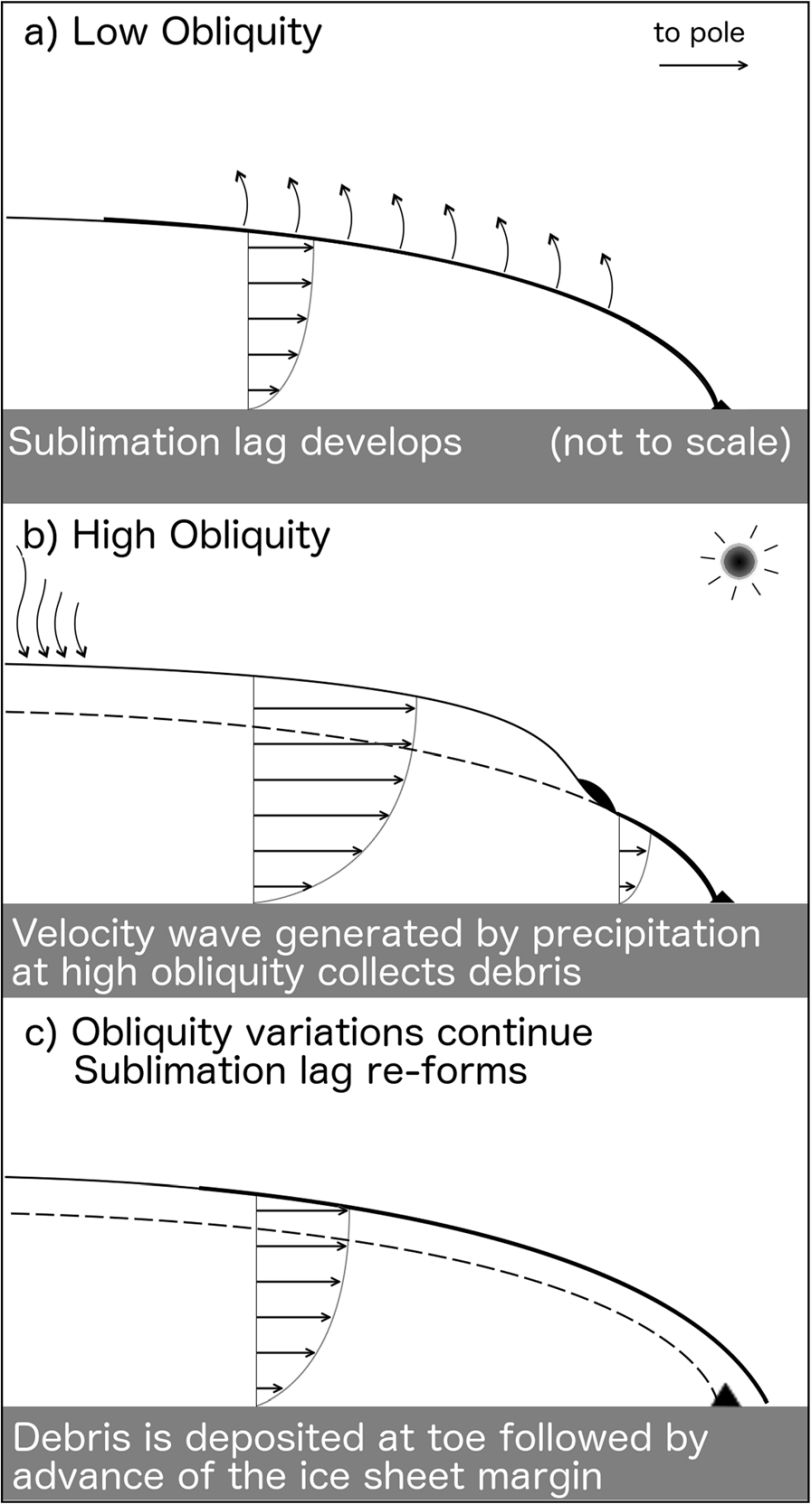
Sketch of proposed mechanism for ridge deposition. **a** Sublimation lag develops on surface of ice sheet. **b** Ice accumulation on the flanks of Pavonis produces a velocity wave which collects surface debris. **c** Debris is deposited at the ice margin and subsequently overridden by the advancing ice sheet

A cartoon depicting our supported hypothesis is shown in Fig. 14. Initial sublimation of a dirty ice deposit results in the formation of a surficial lag layer (Fig. 14a). During high obliquity, accumulation of ice produces a kinematic wave which accumulates surface debris (Fig. 14b) and subsequently deposits it at the ice margin as the ice sheet advances (Fig. 14c). This process would then repeat during the next obliquity cycle. The spacing between ridges would depend on the timing and magnitude of ice accumulation events which dictate how quickly the ice margin advances. Rapid advance would ensue soon after multiple ice accumulation events but would exponentially decrease over time as shown in Fig. 10.

After deposition, the ridges would have become over-ridden by the advancing ice but would have remained preserved due to the lack of basal sliding (as indicated by intersecting ridges such as those in Fig. 1c and the superposed smooth unit on ridges in Fig. 1d). We conclude that the simulation results support an alternative interpretation of ridge sequences as processional (as opposed to recessional) drop moraines.

### Model limitations

The simulation results are subject to our choice of model inputs and the assumptions made within PISM. Our mass balance and temperature calculations are supported by GCM results (Forget et al. 2006) and insolation calculations (Schorghofer 2008; Ward 1974) for Amazonian Mars. The choice to use the recent obliquity history stems from uncertainties in the Martian obliquity prior to 26 Myr ago, so we are focusing on the best constraints currently available for the Amazonian climate. Because ice in our simulation does not reach the full extent of the FSD, there was likely a longer episode, or more frequent episodes, of ice accumulation earlier in the Amazonian. Alternatively, the ablation rate may have been lower than the ~ 0.5 mm/year rate associated with a 0.5 m thick debris layer used in our simulation.

However, our goal is not to reproduce the extent of the FSD, but to simulate the process by which ridges interior to the FSD were deposited. By focusing the past 26 Myr, we are able to show that known climate variations can plausibly result in ridge deposition, although the ridges themselves may be older than this recent obliquity history.

### Basal heat flux and heat transport

Because the Tharsis region is likely subject to a higher than average heat flux, the model assumes a value of 30 mW m^− 2^ based on thermal models presented in Plesa et al. (2016). The primary influence of the basal heat flux is on the temperature at the base of the ice sheet which acts to control the ice sheet thickness. In our simulations, higher heat fluxes result in warmer basal temperatures and more rapid ice deformation which produces a more extensive and thinner ice sheet. Identical Pavonis simulations run with a fixed ice volume using heat fluxes of 20 and 50 mW m^− 2^ give ice thicknesses and extents which are modified by a factor of 0.85 and 1.1, respectively after flow has nearly ceased (after 3 Myr) for the higher heat flux case. Therefore, the simulation presented here (e.g., Fig. 9) may be biased toward slightly slower flow and a somewhat thicker ice sheet. Thinner ice sheets will respond even more dramatically to the obliquity-driven temperature signal because periodic temperature variations tend to become dampened with depth (for example, Fig. 10 and Grott et al. 2007). Therefore, the conclusions regarding the ridge deposition process inferred from the simulation presented here would still apply to a somewhat thinner ice sheet resulting from a higher basal heat flux.

Although PISM provides important insights into the dynamics of Martian ice sheets and captures much of the essential physics, there are aspects of the Martian environment that are not fully represented in the model. For instance, the model assumes that lateral heat transport is dominated by advection and that conduction in the horizontal direction is negligible. An ice thermal conduction lengthscale $\left(d=\sqrt{kt/\left(\rho {c}_p\right)}\right)$ of 2.4 km is associated with an obliquity timescale (*t*) of 120 kyr. Dividing the lengthscale by the timescale gives an estimate for the velocity of a thermal pulse through a Martian ice deposit (equal to 2 cm/year) which is similar to modeled ice flow rates (e.g., Fig. 11b, e). While it is reasonable to neglect heat conduction in the horizontal direction for a terrestrial ice sheet where ice velocities are high (in part due to sliding of ice across the bed), the combination of slow flow and a higher ice thermal conductivity (Eq. 4) at the low temperatures on Mars make the heat flux in the horizontal direction via conduction comparable with that of advection based on the rough calculation above.

### Ice surface temperature and mass balance

Secondly, the surface temperature model inputs rely on the slope of the basal topography rather than the time-varying ice surface topography. Therefore, the influence of the changing ice surface slope on the surface temperature is not captured by the simulation and may be important at the toe of the ice sheet where slopes are steep and pole-facing (in the case of the Pavonis FSD). In particular, the influence of the surface slope on ice temperature may help explain the larger size of ridges found within the Pavonis FSD. As ice advanced northward across the Pavonis FSD, ice temperatures at the toe may have varied markedly as the angle of incident sunlight changed with obliquity—resulting in more accentuated episodes of advance during high obliquity followed by a cold, stagnant margin during low obliquity. In this respect, PISM may be better suited to simulating the westward ice flow which occurred in the Arsia FSD where the margin slope orientation would have less influence on temperature which may account for the smaller, more closely spaced ridges found there.

The surface temperature also plays a key role in determining the ice sublimation rate (Eqs. 5 and 6). As noted in the ice ablation section, the low (~ 0.5 mm/year) ice sublimation rates calculated in our numerical simulation is associated with a debris cover thickness of 0.5 m. If, however, the ice temperature increased from 215 to 220 K, the debris thickness required to maintain a 0.5 mm/year sublimation rate would be 0.9 m. If, however, the debris thickness is maintained at 0.5 m, a 5 K temperature increase (to 220 K) would nearly double the sublimation rate. Therefore, although the simulation rate is nearly constant, an increase in ice loss is likely during times of high obliquity when the pole-facing ice margin is warm. What is uncertain is whether the influence of temperature on sublimation would outweigh the influence of relative humidity which one might expect to be higher during the redistribution of polar ice at high obliquity. It is possible that the increase in sublimation caused by warm temperatures on the pole-facing ice margin during high obliquity is canceled by the reduction in the rate of sublimation caused by an increase in humidity (Fig. 5b). The complicated relationship between obliquity and spatial variations in the mass balance of equatorial ice on Mars is an important topic for future research. In general, increasing the average sublimation rate in our simulations results in fewer ridges which are in close proximity to the distal edge of the accumulation zone (assumed to be 5 km elevation) while lower sublimation rates produce a longer-lived, more extensive ice deposit which can produce more ridges.

Disregarding other factors (atmospheric deposition of volcanic ash or dust) that may influence the debris layer thickness, the factor of ~ 20 increase in particle concentration produced from accumulating surface debris in a kinematic wave suggests that the original sublimation lag thickness can be very roughly estimated by dividing the height of the ridge by 20. However, this “particle concentration factor” will likely change depending on location—increasing as the ice sheet becomes more extensive and debris can be collected over a larger area. Most interior ridges have heights of < 40 m suggesting an original surface debris thickness of < 2 m which would take less than 200 kyr to form from the sublimation of a 5 vol% dusty ice deposit (Fig. 7a). Subsequent to surface lag formation, renewed ice accumulation resulted in advance of the glacial front and ridge deposition. Note that our conclusion that ridge deposition occurs during glacial advance does not run counter to the geologic interpretations of Shean et al. (2005), but does indicate that ice retreat is not needed to form the ridges.

## Conclusions

Thermomechanical ice sheet simulation of the Amazonian Pavonis ice sheet using obliquity inputs from the past 26 Myr provides important insights into the formation of a series of ridges in the interior of the Pavonis FSD. Our simulation assumes an obliquity-precipitation relationship given in Fig. 5 at elevations between 5 and 12 km and ablation rates near 0.5 mm/year in places where ice is not accumulating. This ablation rate is associated with ice buried by a debris lag ≈ 50 cm thick which would form in less than 1 Myr from the sublimation of ice containing > 0.1 vol% dust under low humidity conditions (Fig. 7). The model calculates heat transport in the vertical direction associated with a basal heat flux of 30 mW m^− 2^, deformation-induced strain heat, and obliquity-driven surface temperature variations while advection accounts for the horizontal heat transport. Horizontal ice flow rates are calculated using Glen’s Law (Eq. 3) under the shallow ice approximation.

The reduced ablation rate used in our simulation is a key departure from previous Tharsis ice sheet simulations performed by Fastook et al. (2008). The low ablation rate we employ results in dramatic glacial advance during times of accumulation at high obliquity which gradually slows, stops, and slowly retreats during extended periods of lower obliquity (Fig. 10). During retreat, ice thicknesses are greatly reduced and are not capable of driving flow resulting in a “dead” ice deposit (Fig. 10d). The surface debris on top this ice would be passively dropped and is unlikely to form the long sets of parallel ridges which define a former ice margin.

Instead, we propose that ridges are deposited during glacial advance when precipitation-induced perturbations to the surface ice velocity (Fig. 12b) concentrate surface debris in front of a kinematic wave (Fig. 13c, d, e). Ridges deposited during advance would become buried by the advancing ice sheet but would remain preserved due to the lack of basal sliding of the ice sheet (see “Results and discussion” section). Observational evidence for ridge preservation below an ice sheet comes from intersecting ridge sequences at Arsia (Fig. 1c) and Pavonis (Shean et al. 2005), as well as the superposition of remnant ice (the smooth unit) on top of ridges (Fig. 2b). The periodicity in the ridge spacing (Fig. 3a) is also indicative of an advancing ice sheet which experiences brief periods of ice accumulation causing the ice margin velocity temporarily increase, then gradually slow as the ice thins during advance. This mechanism for ridge emplacement is contrary to the recessional drop moraine hypothesis previously ascribed to these ridges (Fastook et al. 2008; Head and Marchant 2003; Kadish et al. 2014; Shean et al. 2005) and suggests that the formation of individual ridges takes place over a shorter timescale than previously thought.

We conclude that initial sublimation of the uppermost portions of the ice deposit likely resulted in the formation of a debris lag which protected ice from further ablation—allowing it to slowly advance and deposit moraines as its flow velocity periodically fluctuated with obliquity. This new interpretation of the FSD ridge formation process provides an opportunity to further constrain the Amazonian climate history of Mars.

## Supplementary information

**Additional file 1: Figure S1.** Obliquity History Five representative scenarios for the obliquity of Mars determined by Laskar et al. 2004 over the past 250 Myrs (a) and 26 Myrs (b). The different colored lines refer to different assumed values for the initial precession rate of the rotation axis of Mars which produces chaotically different results over long timescales (b), but nearly identical results in the past 26 Myrs (a). Thick lines give obliquity over a 1 Myr averaging window. Scenario "P000" is used as our model input.

**Additional file 2.** Pavonis Simulation 20 mW/m2. Animated plots of the ice flow simulation which uses a basal heat flux of 20 mW/m^2^ showing the extent, thickness, basal stress, approximate heat flux, ice temperature, ice effective viscosity, and ice surface velocity.

**Additional file 3.** Pavonis Simulation Earth Gravity 20 mW/m2. Animated plots of the ice flow simulation using Earth-like gravity and a basal heat flux of 20 mW/m^2^ showing the extent, thickness, basal stress, approximate heat flux, ice temperature, ice effective viscosity, and ice surface velocity.

**Additional file 4.** Pavonis Simulation 30 mW/m2. Animated plots of the ice flow simulation presented in the text which uses a basal heat flux of 30 mW/m^2^ showing the extent, thickness, basal stress, approximate heat flux, ice temperature, ice effective viscosity, and ice surface velocity.

## Data Availability

Simulation results and CTX stereo elevation data is available upon request from RAP.

## Abbreviations

CTX: Context camera
DEM: Digital elevation model
FSD: Fan-shaped deposit
MOLA: Mars Orbiter Laser Altimeter
PISM: Parallel ice sheet model
