# Supplementary figures and images for: Cold-based glaciation of Pavonis Mons, Mars: evidence for moraine deposition during glacial advance
Source: Prog Earth Planet Sci. 2020 Mar 12;7(1):13. doi: 10.1186/s40645-020-0323-9 (PMC7194259; doi:10.1186/s40645-020-0323-9)

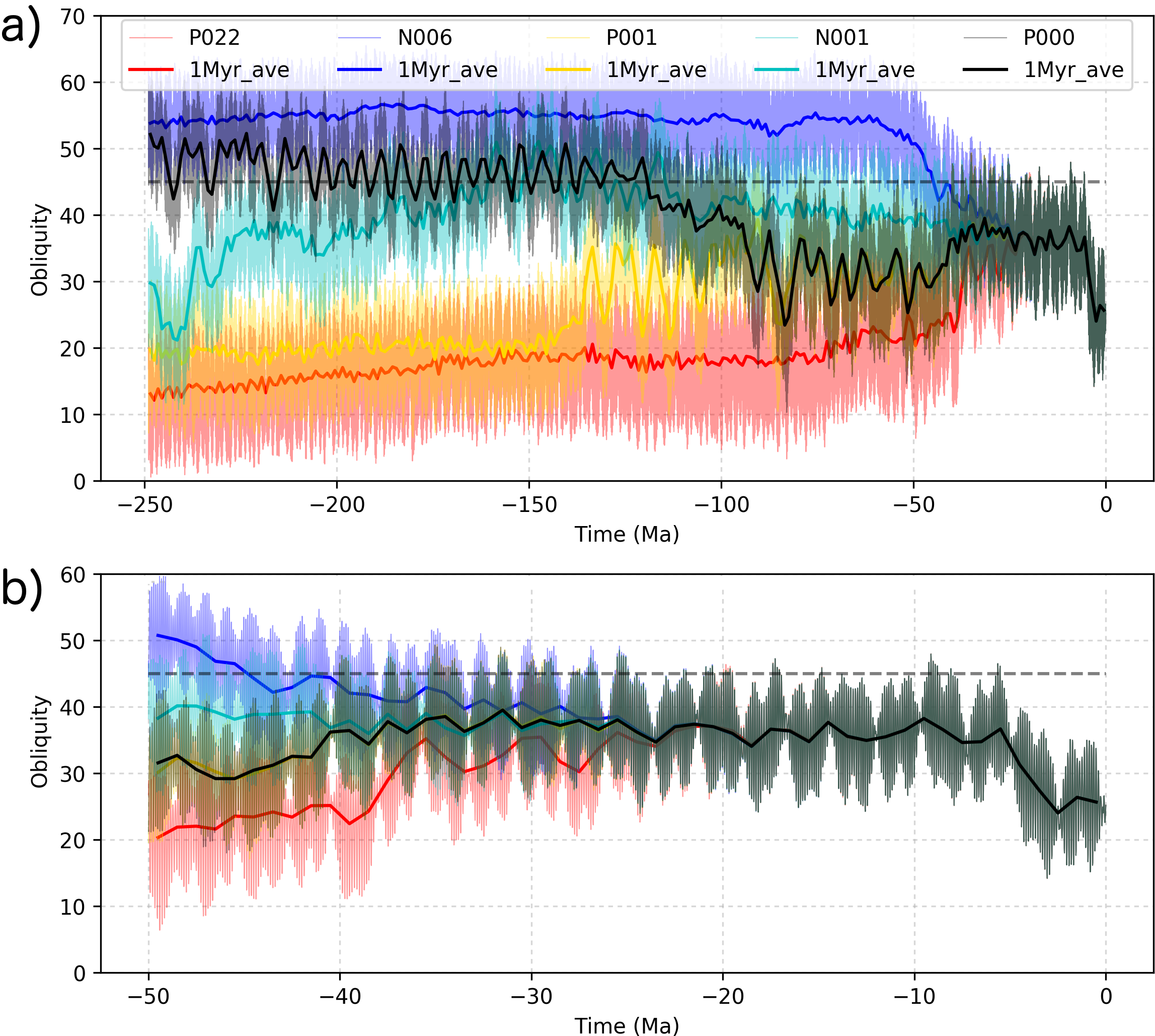

Supplement: Supplementary file 1 — Additional file 1: Figure S1. Obliquity History Five representative scenarios for the obliquity of Mars determined by Laskar et al. 2004 over the past 250 Myrs (a) and 26 Myrs (b). The different colored lines refer to different assumed values for the initial precession rate of the rotation axis of Mars which produces chaotically different results over long timescales (b), but nearly identical results in the past 26 Myrs (a). Thick lines give obliquity over a 1 Myr averaging window. Scenario "P000" is used as our model input. [file 40645_2020_323_MOESM1_ESM.png]
